# DNA damage and transcriptional regulation in iPSC-derived neurons from Ataxia Telangiectasia patients

**DOI:** 10.1038/s41598-018-36912-0

**Published:** 2019-01-24

**Authors:** Alessandro Corti, Raina Sota, Matteo Dugo, Raffaele A. Calogero, Benedetta Terragni, Massimo Mantegazza, Silvana Franceschetti, Michela Restelli, Patrizia Gasparini, Daniele Lecis, Krystyna H. Chrzanowska, Domenico Delia

**Affiliations:** 1 Department of Research, Fondazione IRCCS Istituto Nazionale Tumori, Milano, Via Amadeo 42, 20133 Milano, Italy; 2 Department of Applied Research and Technological Development, Fondazione IRCCS Istituto Nazionale Tumori, Via Amadeo 42, 20133 Milano, Italy; 3 Universita’ degli Studi di Torino, Bioinformatics and Genomics Unit, Molecular Biotechnology Centre, Via Nizza 52, 10126 Torino, Italy; 4 Fondazione IRCCS Istituto Neurologico Carlo Besta, Department of Neurophysiopathology and Diagnostic Epileptology, Via Celoria 11, 20133 Milano, Italy; 5 Institute of Molecular and Cellular Pharmacology (IPMC) LabEx ICST, CNRS UMR7275, Route des Lucioles, 06560 Valbonne, Sophia Antipolis France; 6 University Côte d’Azur, 660 Route des Lucioles, 06560 Valbonne, Sophia Antipolis France; 7 Department of Research, Fondazione IRCCS Istituto Nazionale Tumori, Via Amadeo 42, 20133 Milano, Italy; 8 Department of Research, Fondazione IRCCS Istituto Nazionale Tumori, Milano, Via G Venezian 1, 20133 Milano, Italy; 9 Department of Medical Genetics, The Children’s Memorial Health Institute, Al. Dzieci Polskich 20, 04-730 Warsaw, Poland; 10 Present Address: IFOM, FIRC Institute of Molecular Oncology, Via Adamello 16, 20139 Milano, Italy

## Abstract

Ataxia Telangiectasia (A-T) is neurodegenerative syndrome caused by inherited mutations inactivating the ATM kinase, a master regulator of the DNA damage response (DDR). What makes neurons vulnerable to ATM loss remains unclear. In this study we assessed on human iPSC-derived neurons whether the abnormal accumulation of DNA-Topoisomerase 1 adducts (Top1ccs) found in A-T impairs transcription elongation, thus favoring neurodegeneration. Furthermore, whether neuronal activity-induced immediate early genes (IEGs), a process involving the formation of DNA breaks, is affected by ATM deficiency. We found that Top1cc trapping by CPT induces an ATM-dependent DDR as well as an ATM-independent induction of IEGs and repression especially of long genes. As revealed by nascent RNA sequencing, transcriptional elongation and recovery were found to proceed with the same rate, irrespective of gene length and ATM status. Neuronal activity induced by glutamate receptors stimulation, or membrane depolarization with KCl, triggered a DDR and expression of IEGs, the latter independent of ATM. In unperturbed A-T neurons a set of genes (FN1, DCN, RASGRF1, FZD1, EOMES, SHH, NR2E1) implicated in the development, maintenance and physiology of central nervous system was specifically downregulated, underscoring their potential involvement in the neurodegenerative process in A-T patients.

## Introduction

Ataxia Telangiectasia (A-T) is an inherited syndrome manifesting early onset neurodegeneration, cancer predisposition, immunodeficiency, telangiectasias, and at cellular level radiosensitivity, chromosomal instability and cell cycle checkpoint defects^1^. Neuropathological abnormalities in A-T include progressive death of cerebellar Purkinje and granular, cells, moderate decay of the bulbar olivae in the brainstem, and mild loss of myelinated fibers in corticospinal and spinocerebellar tracts^2^. A-T is caused by loss of function mutations in *ATM* gene, which encodes a protein kinase acting in the nucleus as the apical sensor and transducer of DNA double strand breaks (DSBs). ATM is rapidly activated by DSBs and recruited at sites of lesions, inducing the phosphorylation at TQ/SQ residues of several downstream proteins implicated in cell cycle checkpoints arrest, repair of DSBs, local chromatin remodelling, apoptosis and senescence^3^. Besides its primary role in DNA damage response (DDR), ATM has been implicated in redox-sensing and proteostasis^4^, mitochondrial homeostasis via mitophagy^5^, autophagy of peroxisomes^6^. Very recently, cytoplasmic ATM was shown to segregate with and regulate endocytosis of synaptic vesicles, as opposite to ATR which associates with inhibitory vesicles^7^.

Currently, it is uncertain what renders neurons hypersensitive to ATM deficiency. In line with the canonical function of ATM in DDR, brain degeneration in A-T has been ascribed to the inappropriate DNA repair in pre- and post-mitotic neurons, boosting the rate of genotoxic lesions. However, increased oxidative stress and reduced anti-oxidant defense, themselves reflecting a non-canonical cytoplasmic activity of ATM, may equally play a role in A-T, considering that high oxygen consumption and metabolic activity expose brain neurons to elevated ROS levels that can induce abundant SSBs^8,9^. Neurodegeneration has also been ascribed to transcriptional decline of genes implicated in synaptic vesicle trafficking and release^10,11^. Interestingly, an age-dependent accumulation of mutations believed to arise during transcription has been detected at single-neuron level in normal individuals and to a greater extent in DNA repair-deficient neurodegenerative syndromes^12,13^. A connection between ATM and Topoisomerase 1 (Top1) in the maintenance of transcription integrity has recently emerged. Top1 relaxes DNA supercoiling generated by transcription, replication and chromatin remodeling via transient formation of Top1-DNA covalent complexes (Top1-cc) and introduction of SSBs. The proteolytic removal of Top1-ccs, an essential step to avoid collision with the replication or transcriptional machinery and conversion of SSBs into lethal DSBs^14^, is ATM-dependent^15^, and the accumulation in ATM deficient cells of Top1-ccs^15,16^ and aberrant DNA lesions^17^ underscores their neuropathogenic relevance in A-T. A recent hypothesis suggests that A-T brain degeneration might arise from dysfunctional glial cells, in turn impairing the functionality and viability of neural cells^18^.

Patient-derived induced pluripotent stem cells (iPSCs) differentiating into mature neurons offer a powerful *in vitro* system to model neurological diseases^19,20^. Using this approach, we investigated the response of A-T and normal (WT) neurons to various stimuli and impact on transcription, to identify factors and mechanisms of neuropathological relevance for A-T.

## Results

### hiPSC-**derived** normal and A-T neurons

The hiPSCs used in this study were established from dermal fibroblasts of two unrelated A-T patients (A-T1 and A-T2) and two healthy controls (WT1 and WT2), as described^16,21^ and further detailed in the Methods and Supplemental data sections. The phenotypic and electrophysiological properties of the hiPSC-derived neurons from WT2 and A-T2 have been reported^16^, while those from WT1 and A-T1 are found in Suppl. Figs 1–4.

### CPT activates an ATM-dependent DDR

We previously reported that human postmitotic A-T neurons exhibit an abnormal accumulation of topoisomerase 1 covalently bound to DNA (Top1-cc)^16^, in agreement with findings in mouse Atm−/− neural and MEF cells^15,17,22^. Top1 activity facilitates transcription by introducing transient breaks on one strand of the DNA especially in gene bodies, which dissipate supercoiling and stimulate RNA Pol II pause release^23,24^. It has also been shown that ATM loss markedly delays transcription recovery following release from Top1-cc trapping^15^.

**Figure 1:**
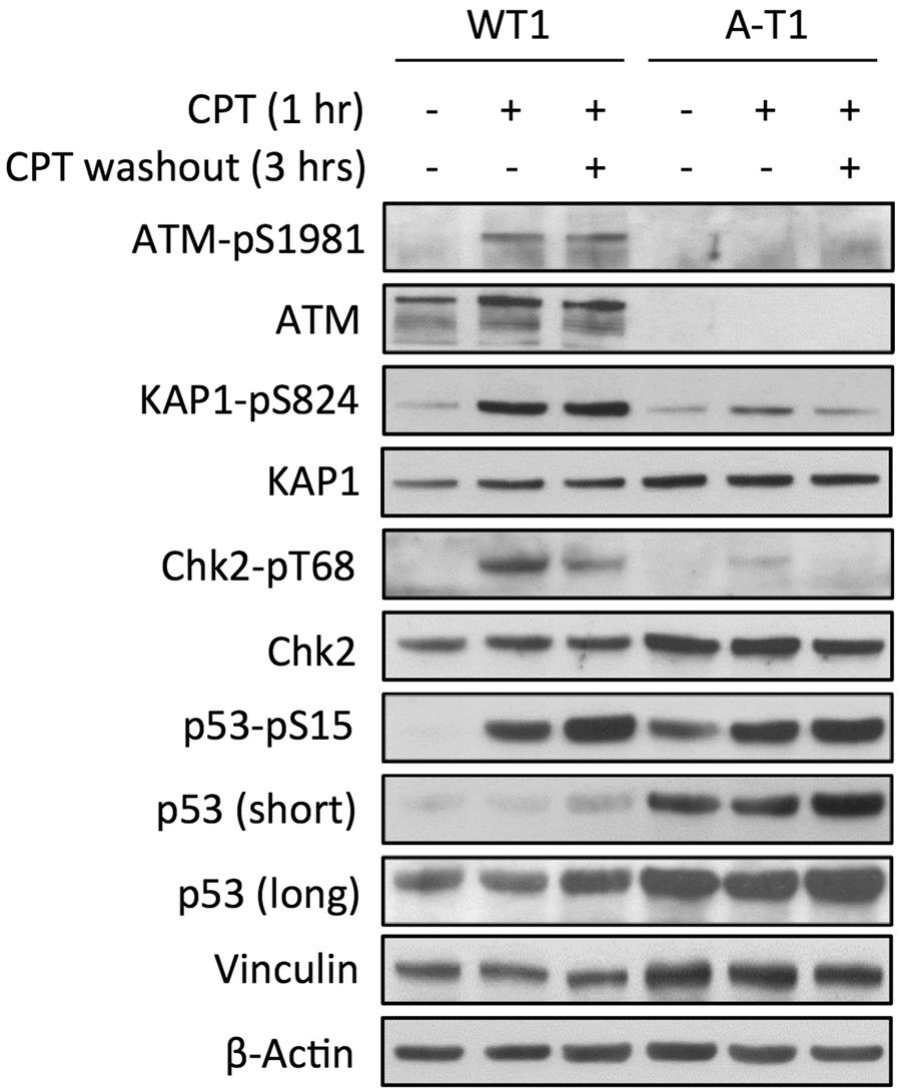
CPT induces the ATM activation and DDR nuclear foci. DIV30 neurons were treated for 1 hr with CPT and collected either immediately or 3 hrs after drug washout. The DDR was evaluated by Western blot using phospho- and pan-specific antibodies. β-Actin and vinculin are protein loading controls.

To determine whether the elevated levels of Top1-cc affected transcription, we first analysed the ATM-dependent DDR in postmitotic neurons treated with CPT for 1 hr, and then allowed to recover for 3hrs after drug washout. This treatment schedule, which was not cytotoxic, according to viability tests and absence of cleaved PARP (not shown), induced in WT neurons the autophosphorylation of ATM on Ser1981 (an indicator of its kinase activity) and phosphorylation of its substrates KAP1-Ser824, Chk2-Thr68 and p53-Ser15, both at 1 hr and 3hrs post-recovery (Fig. 1), whereas in A-T neurons these modifications were markedly attenuated. The DNA damaging effect of CPT in WT neurons resulted in the formation at 1 hr of 53BP1 nuclear foci (Suppl. Fig. 5a), most being resolved during the next 3 hrs, while in A-T these foci were less evident, concordant with the fact that ATM deficiency attenuates the DDR. The DSB-inducing agent neocarzinostatin (NCS) used as positive control, gave similar results (Suppl. Fig. 5a).

### CPT-induced transcriptional changes, and correlation with gene length

**Figure 2:**
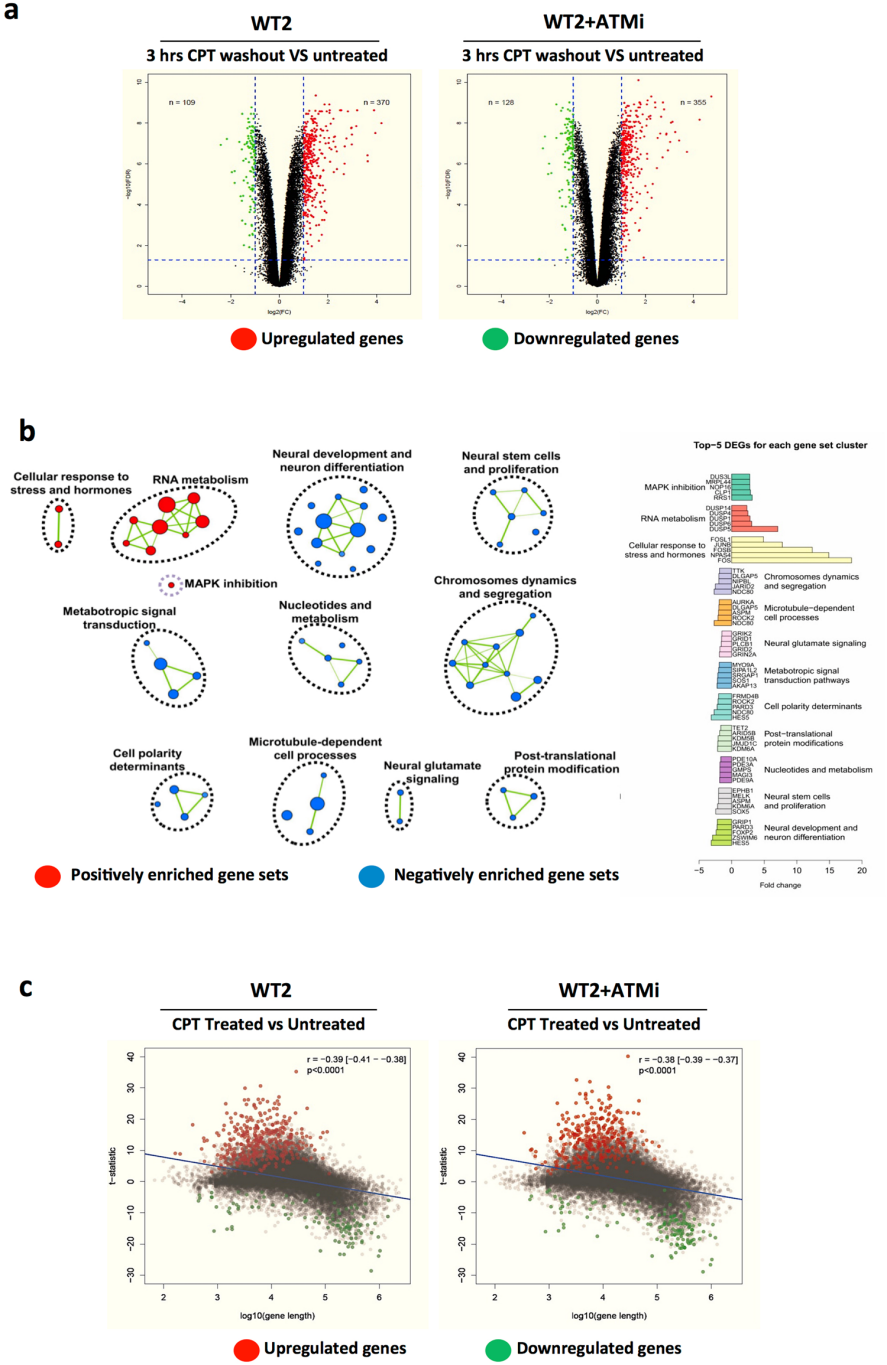
CPT treatment affects global gene expression in neurons. DIV30 WT2 neurons were untreated or treated for 1 hr with CPT and harvested 3hrs after drug washout; the ATM inhibitor was added 1 hrs prior to CPT. Gene expression and alternative splicing of the samples was analysed using the gene chip human exon array. (**a)** Volcano plots depicting the log2 fold change (FC, X-axis) of the significantly up- and down-regulated genes in relation to log10 false discovery rate (FDR, Y axis). The number of CPT-modulated genes is indicated in the left (FC ≤ −2 and FDR < 0.05) and right (FC ≥ 2 and FDR < 0.05) quadrants of the plots. (**b)** Interaction nodes and clusters for the differentially expressed positively and negatively enriched gene sets (FDR < 0.005), generated from WT2 (untreated vs CPT treated) using Cytoscape. On the right are reported the top five DEGs for each cluster’s assigned biological function. (**c)** Scatter plot of the correlation (R) between the log10 gene length (X axis) and the t-statistic (Y axis) for the up- and down-regulated genes.

As Top1 inhibitors in mouse neurons repress the global gene expression^25,26^, we performed exon microarray analysis to determine the transcriptional impact of Top1-cc blocking on WT2 neurons pretreated or not with the ATM inhibitor Kudos-55933 (WT2-ATMi). The ablation of the CPT-induced phosphorylation of ATM substrates validated the effectiveness of the inhibitor (Suppl. Fig. 5b). CPT treatment caused in WT2 neurons the upregulation of 370 genes and downregulation of 109, while in WT2-ATMi the upregulation of 355 genes and downregulation of 128 (Fig. 2a and Suppl. Table 1 for the full list of modulated genes**)**. GSEA analysis of WT2 neurons showed, for the upregulated genes, enrichment for “cellular response to stress and hormones” and “RNA metabolism” pathways, and for the downregulated genes enrichment for pathways involved in”neural development and differentiation”, “metabotropic signal transduction”, “nucleotides and metabolism“ (Fig. 2b and full list in Suppl. Table 2).

Since Top1 inhibition in mouse neurons suppresses the expression of long genes by impairing transcription elongation^23,25,26^, we determined the correlation among the CPT-modulated genes with gene length. We found that the length of the downregulated genes was on average significantly greater (175 Kb) than those upregulated (125 Kb) in both WT2 and WT2-ATMi groups (Fig. 2c).

### CPT induces the expression of immediate early genes (IEGs) irrespective of ATM

**Figure 3:**
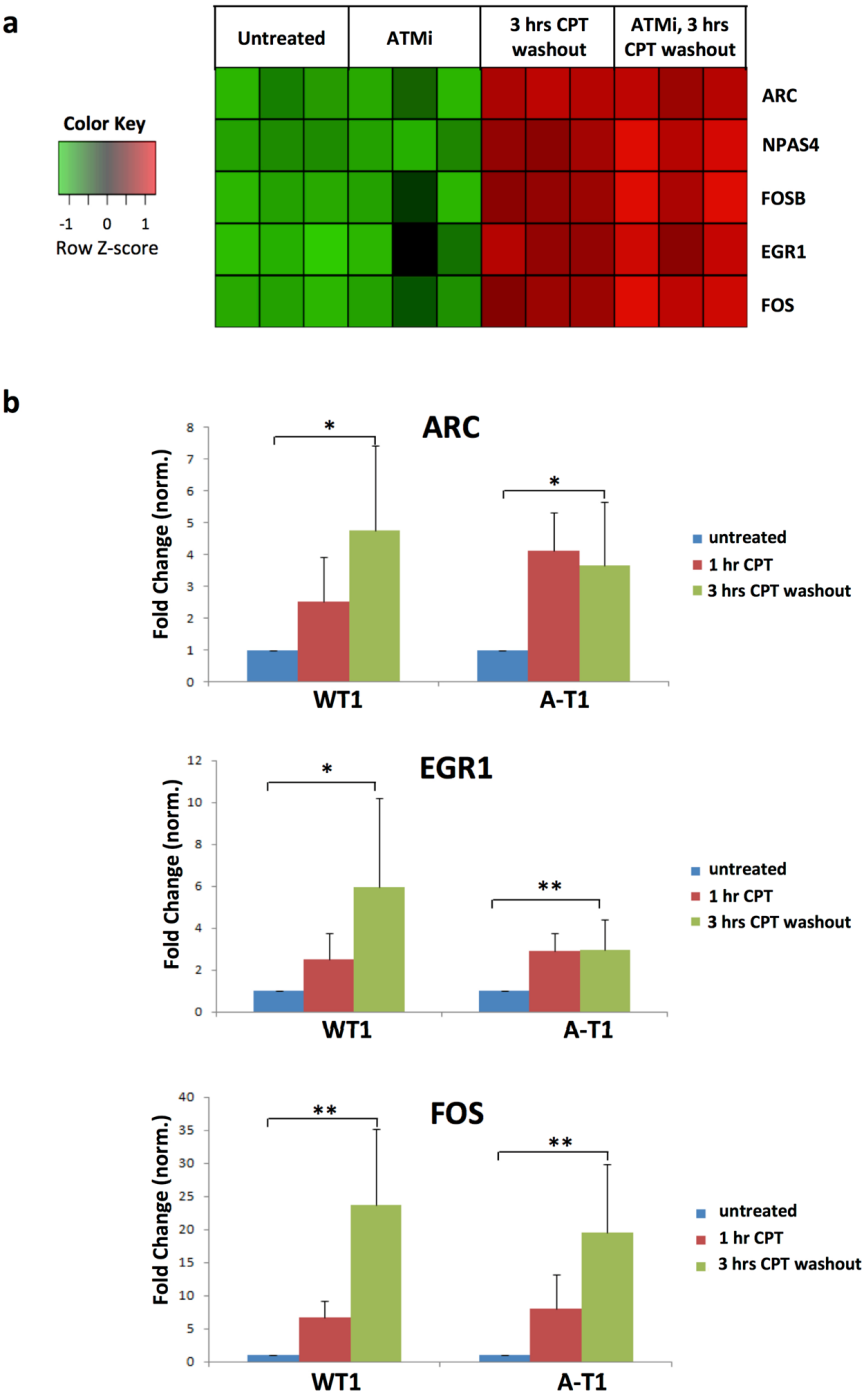
IEGs are transcriptionally induced by CPT treatment. DIV30 neurons were treated and subjected to exon array analysis as in Fig. 2. (**a**) Heat map depicting the expression of IEGs induced by CPT, in relation to ATM activity. (**b)** qRT-PCR analysis of ARC, EGR1, FOS and GAPDH (the latter used for normalization) performed on mRNA extracted from WT1 and A-T1 neuronal preparations. Data are average of six experiments +/−SD and results normalized against those of GAPDH (* p < 0.05; **p < 0.01).

**Figure 4:**
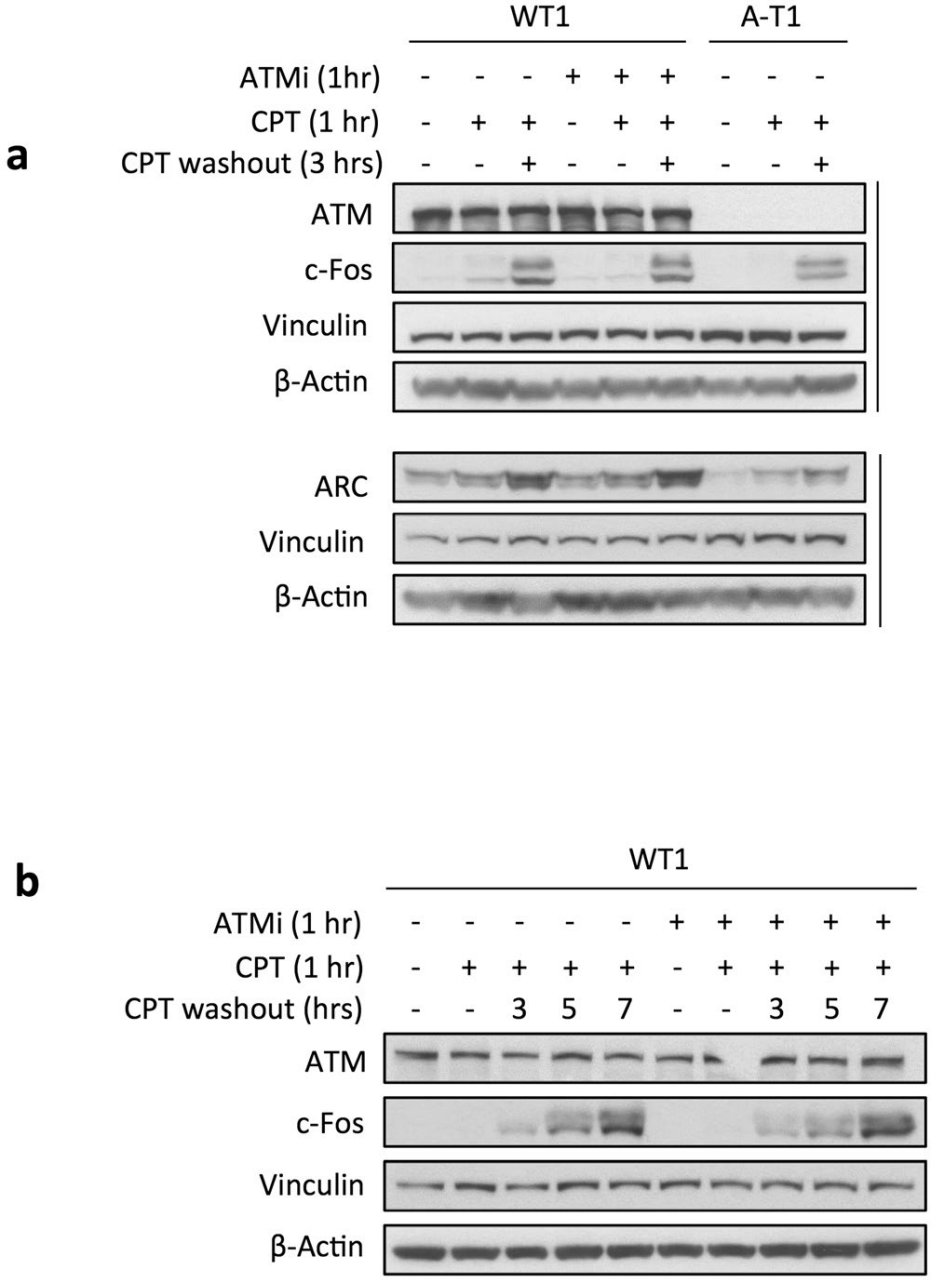
CPT induces the protein expression of IEGs. Western blots were performed on DIV30 neurons treated for 1 hr with CPT and collected immediately and 3hrs (**a**) or at later time (**b**) after drug washout. The ATM inhibitor was added 1hr prior CPT and left throughout the experiment. Vinculin and β-Actin are loading controls for proteins.

The exon array analysis also revealed that CPT upregulated in WT2 and WT2-ATMi neurons a number of IEGs such as NPAS4, FOS, FOSB, EGR1 and ARC **(**Fig. 3a**)**, many of which encode transcription factors^27^. In the CNS, IEGs are rapidly induced by synaptic activity, promoting in turn the expression of late-response genes, eg: Bdnf and Nptx2, that are crucial for synaptic plasticity, learning and memory^27,28^. The exon microarray findings were validated by qRT-PCR assays on separate cultures of WT1 and A-T1 neurons, and in all cases CPT upregulated the IEGs (Fig. 3b), confirming the ATM-independence of this response. Furthermore, the accumulation of ARC and c-Fos proteins in WT1, WT1-ATMi and A-T1 neurons became evident at 3hrs post-recovery from CPT treatment and persisted up to 7hrs (Fig. 4a,b).

### Transcription elongation in WT and ATM-deficient neurons

**Figure 5:**
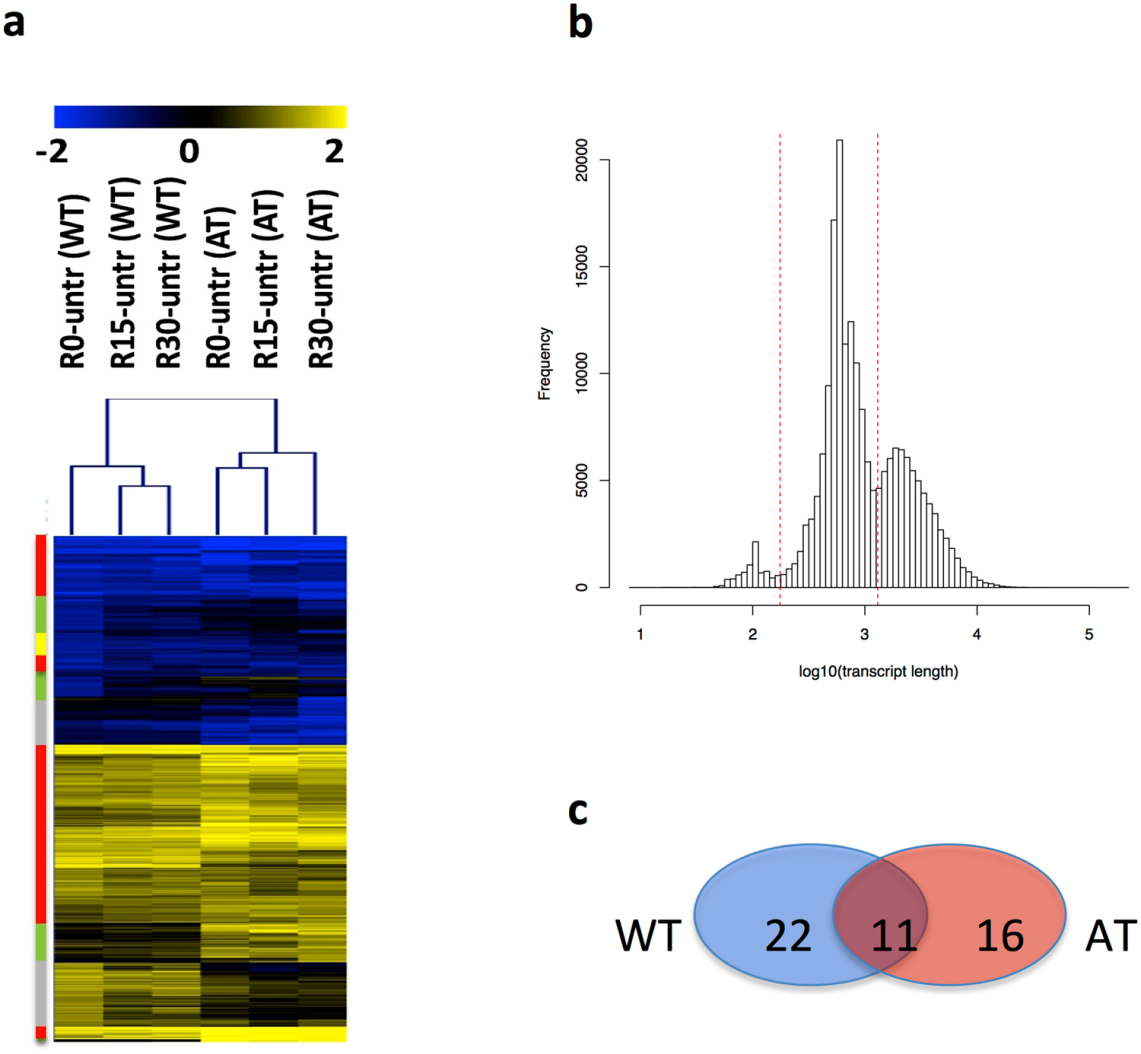
Hierarchical clustering analysis of differential expression at transcripts and exons level. (**a)** Log_2_ expression ratio between CPT treated and untreated cells for the 5666 transcripts detected as differentially expressed in at least one of the two experimental groups, i.e. WT2 and A-T2. (**b)** ENSEMBL transcripts distribution as function of transcripts’ length. Dashed lines indicate the three groups of transcripts, from left to right, ≤175 nts (7872 transcripts), (ii) >175 nts ≥1300 nts (122719 transcripts) ad (iii) >1300 nts (67307 transcripts). (**c)** Transcripts showing a Pearson correlation below 0.2, in all replicates, comparing CPT treated vs untreated WT2 and A-T2 neurons.

Topoisomerases are indispensable in transcription and their deregulation is associated with neurodegenerative diseases^14^. Specifically, Top1 deletion represses both in mouse and human neurons the expression of extremely long genes^23,25^. The evidence that ATM-deficient cells overexpress Top1-ccs^15,16^ raised the question whether this abnormality might affect transcription elongation in neurons. To verify this possibility, we performed RNAseq analysis on nascent RNA, purified as detailed in Methods, from WT2 and A-T2 neurons that were: i) untreated (Untr); ii) treated with CPT for 1 hr (R0); iii) treated with CPT for 1 hr and cultured in drug-free medium for 15 min (R15) or 30 min (R30). Transcripts differentially expressed between R0 and Untr conditions accounted for 3813 in WT2 and 3397 in A-T2, for a total of 7210 transcripts of which 1544 were common to both groups while the remaining 5666 were not. To depict changes in transcription rescue between WT2 and A-T2 neurons, hierarchical clustering analysis was done on the 5666 differentially expressed transcripts (Fig. 5a and Suppl. Table 3). Hierarchical clustering highlighted different expression patterns: i) genes with a similar profile in WT2 and A-T2, independent of the treatment conditions (Fig. 5a, red cluster); ii) genes showing an increased expression in A-T2 compared to WT2 (Fig. 5a, green cluster); iii) genes with an increased expression only in A-T2 upon removal of CPT (Fig. 5a, yellow cluster); iv) genes with a reduced expression in A-T2, irrespective of the treatment conditions (Fig. 5a, grey cluster). However, none of the expressed genes in A-T2 selectively showed a reduced efficiency in transcription rescue compared to WT2.

The potential differences between A-T2 and WT2 at the level of efficiency of transcription recovery in long transcripts were further investigated using transcripts coverage, which provides for a given transcript a profile of the exons expression and position. Coverage analysis was used to estimate if asymmetries in transcription rescue efficiency could be detected between A-T2 and WT2 upon CPT washout. In brief, ENSEMBL transcripts (197898) were divided into three length groups (Fig. 5b): (i) ≤175 nts (7872 transcripts), (ii) >175 nts ≥1300 nts (122719 transcripts) ad iii) >1300 nts (67307 transcripts). Coverage comparison analysis was done only for transcripts longer than 1300 nts. Coverage was calculated for these transcripts in each experimental specimen, and then Pearson correlation between R0 and Untr was determined in A-T2 and WT2 samples. Only transcripts showing coverage profile changes induced by CPT were retained for the subsequent analysis, i.e. Pearson correlation <0.2. Coverage profile changes were observed in 27 A-T2 and 33 WT2 transcripts, of which 11 transcripts were in common (Fig. 5c). Thus, the total number of transcripts, in which coverage is affected by CPT treatment in A-T2 or WT2 are 49. Dissimilarities in coverage profiles between A-T2 and WT2 were investigated on these 49 transcripts comparing the coverage in Untr with respect to R15 or R30 specimens. Coverage dissimilarity was estimated using the integrative correlation analysis (ICC)^29^. ICC was developed for quantification of the extent to which independent studies can be reliably analyzed together for the systematic comparison of transcription profiles^30^. This analysis did not detect any consistent change between A-T2 and WT2 groups (not shown).

### Neuronal activity induces an ATM-dependent DNA damage response

**Figure 6:**
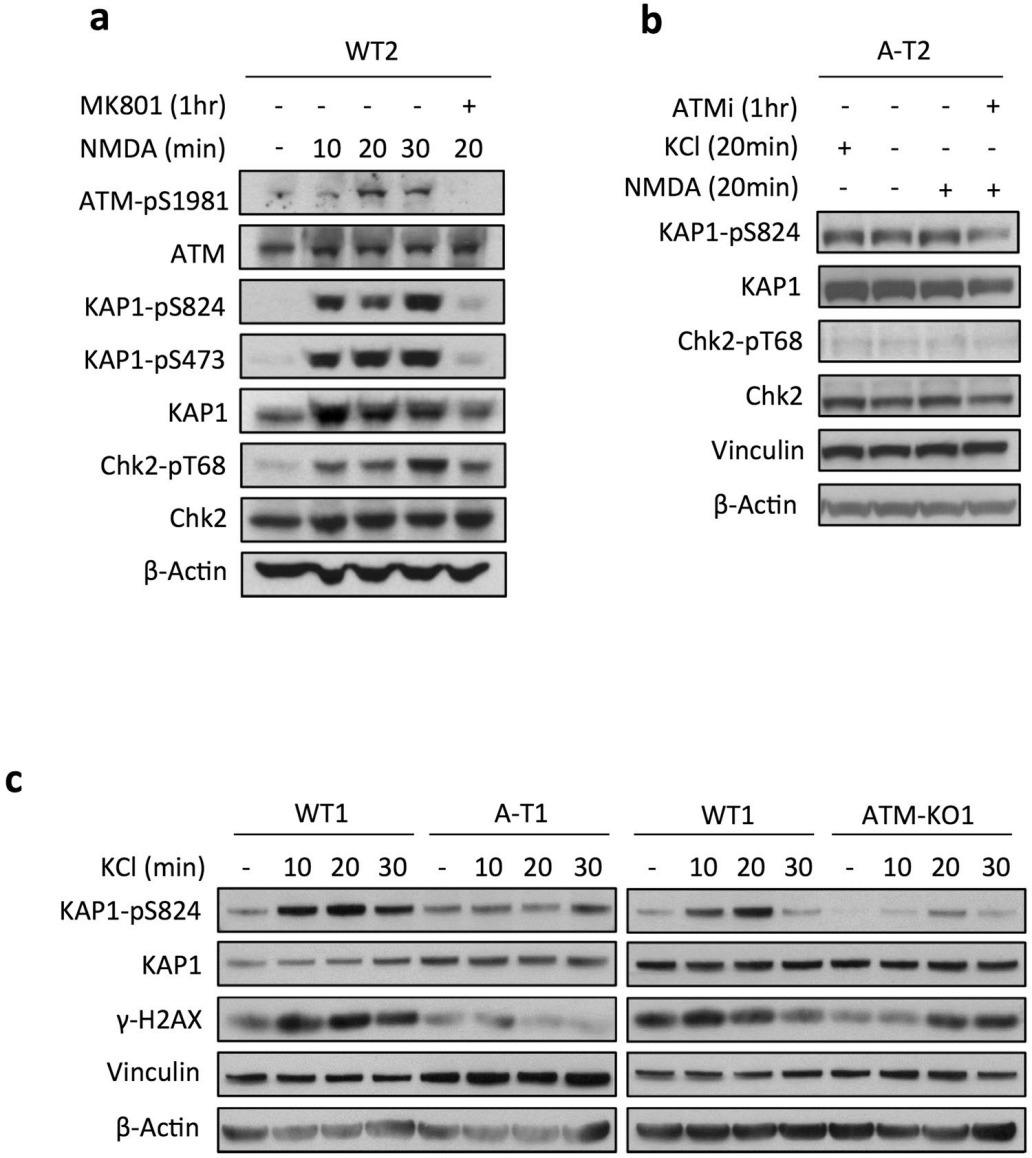
Physiological stimulation of neuronal activity induces a DDR signalling. DIV55 neurons were treated with NMDA or KCl for up to 30 min and analysed for the phosphorylation of ATM targets. To inhibit action potential, neurons were pre-incubated overnight with the sodium channel blocker TTX. (**a)** Time-dependent phosphorylation of ATM targets induced by NMDA. Pre-incubation for 1 hr with the NMDA receptor antagonist MK801 was also performed (lane 5). (**b)** The ATMi was added 1 hr prior to treatment for 20 min with NMDA or KCl. (**c)** Time-dependent phosphorylation of ATM targets upon KCl-depolarization. Vinculin and β-Actin are loading controls for proteins.

Synaptic activity in murine neurons elicits a rapid phosphorylation of proteins at S/TQ, a typical consensus site for ATM and other PIKK family kinases^31^. To find out whether human neurons exhibit a similar response, electrophysiologically active DIV55 neurons (see above and ref 16) were treated with AMPA and NMDA to stimulate ionotropic glutamate receptors, or depolarized with KCl, and assessed for ATM activity. Immunoblot analysis of NMDA-stimulated WT2 neurons showed highest levels of ATM-Ser1981 autophosphorylation and phosphorylation of its substrates KAP1-Ser824 and Chk2-Thr68 between 20 and 30 min (Fig. 6a, lanes 3–4), a response abolished by pre-treatment with the NMDA receptor antagonist MK801 (Fig. 6a, compare lanes 3 and 5), demonstrating the NMDA receptor specificity. Conversely, no ATM signalling was detected in NMDA-treated A-T2 and WT2-ATMi neurons (Fig. 6b), highlighting the ATM-dependence of this phenomenon. AMPA, like NMDA, induced in WT2 neurons an ATM response within 20 min (Suppl. Fig. 6). Similarly, KCl-induced membrane depolarization, elicited an ATM signalling in WT1, but not A-T1 or ATM-KO1 neurons (Fig. 6c), the latter derived from WT1, in which the ATM gene was knockout by CRISPR (Suppl. Fig. 7 for characterization of the clone).

Various paradigms of neuronal stimulation *in vivo* or in culture have shown to induce DSBs^32,33^ found to map within promoters of IEGs and to be indispensable for their transcription^33,34^. DNA breaks and DDR play also a positive role in transcriptional elongation of stimulus-inducible genes^35^. To determine whether DNA breaks also occur in activity-stimulated human neurons, we analysed the phosphorylation of H2AX-Ser139 (γ-H2AX), a target of ATM and known biomarker of DSBs, and found increased levels of γ-H2AX by already 10 min of depolarization with KCl in WT1, but not ATM-KO1 or A-T1 neurons (Fig. 6c), and a similar response was evoked by NMDA or AMPA stimulation (not shown). Altogether, these findings indicate that human neuronal activity initiates an ATM signalling cascade, most likely emanating from DNA damage.

### Neuronal activity induces the expression of IEGs independent of ATM

**Figure 7:**
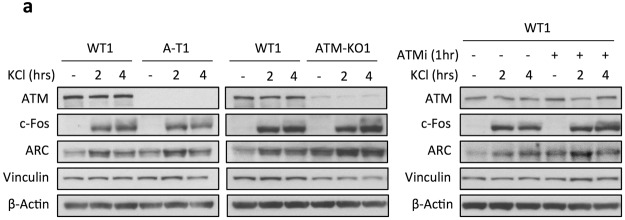
Neuronal activity enhances the expression of ARC and c-Fos proteins DIV55 neurons were incubated overnight with TTX, thereafter with KCl for up to 4hrs and assessed by Western blot for c-Fos and ARC proteins. Note the ATM-independent induction of these IEGs, as also evident from the WT1 treated with ATMi (right panel). Vinculin and β-Actin are loading controls for proteins.

To determine whether physiological stimuli induce a similar expression of the IEGs as CPT, we analysed the levels of c-Fos and ARC after treatment of with KCl, and found an accumulation of these proteins by 2hrs of depolarization, both in WT1, A-T1 and ATM-KO1 neurons (Fig. 7). The induction of IEGs in WT1 neurons was not prevented by pre-treatment with ATMi.

**Figure 8:**
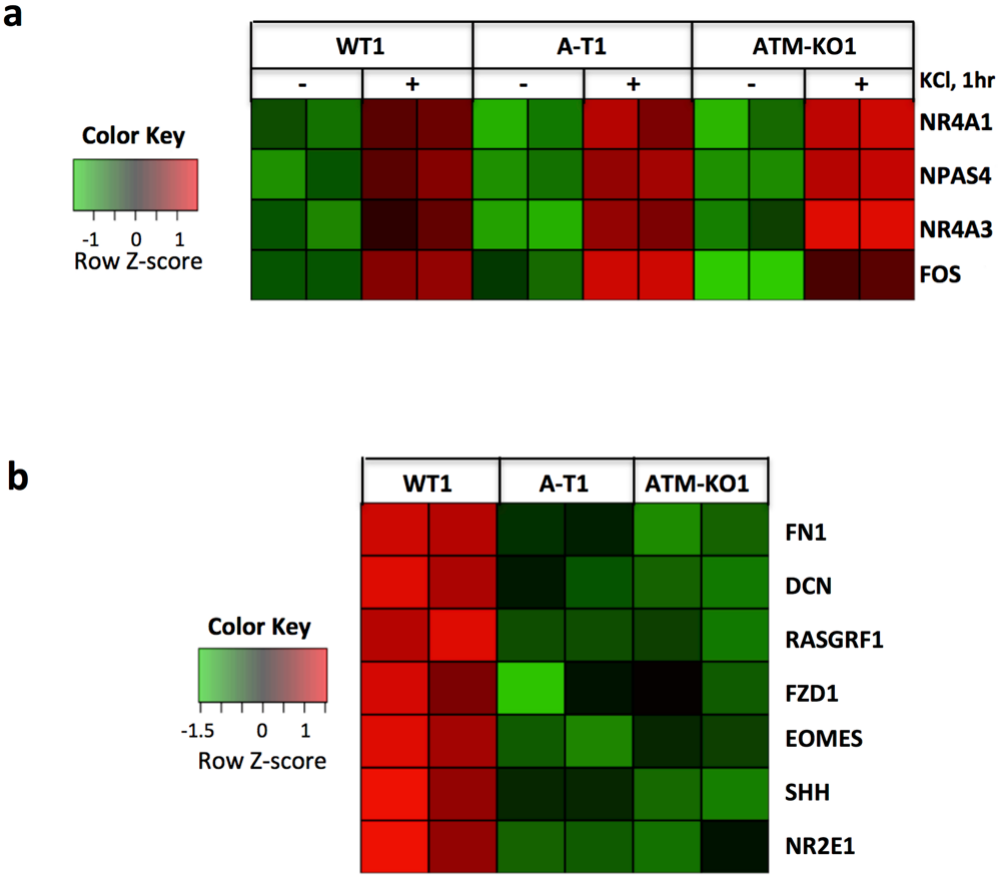
Neuronal activity-induced IEG and basal transcriptional expression. WT1, A-T1 and ATM-KO neurons at DIV55 were incubated overnight with TTX and then untreated or treated with KCl for 1 hr, harvested and analysed by RNA-seq. (**a)** Heat map of the stimulus-induced IEGs. (**b)** heat map of genes differentially expressed in unperturbed WT1 compared to A-T1 and ATM-KO1 neurons. These genes are involved in CNS development, maintenance, and synaptic plasticity. Data are of two independent replicates.

To identify transcriptomic differences in relation to neuronal activity and ATM expression, we performed RNA-Seq analysis on WT1, A-T1 and ATM-KO1 neurons treated or untreated with KCl for 1 hr. A group of differentially expressed up- and down-regulated genes (DEGs) was identified for each neuronal line following KCl-depolarization (Suppl. Table 4). The representation of these DEGs as Venn diagram (Suppl. Fig. 8) allowed us to define genes shared between WT1, A-T1 and ATM-KO1 neurons. Specifically, a small set of genes that included NR4A3, FOS, NR4A1 and NPAS4 (Fig. 8a), was strongly upregulated after treatment in all three cell lines. These genes belong to the IEGs family^27,36,37^, typically induced by depolarizing stimuli *in vitro* and sensory stimuli *in vivo*^28^. Concordant with the immunoblots (Fig. 7a), the RNA-Seq data confirm that the physiological induction of IEGs, such as FOS, is mainly ATM independent. RNA-Seq data were validated by RT-PCR analysis of the FOS transcript in treated and untreated samples (Suppl. Fig. 9). Of note, PPP1R15A, a gene implicated in DDR and cellular stress^38^, was transcriptionally induced only in stimulated WT1 neurons (Suppl. Table 4). This specific induction might be explained by the activation of the DDR elicited by the accumulation of activity-dependent DSBs, which is known to be defective in A-T cells.

### A-T and ATM-KO neurons share significant differences in the basal transcriptomic profile compared to WT neurons

To find out if ATM deficiency affects the basal transcriptional signature, we compared the RNA-Seq profiles of the unperturbed WT1, A-T1 and ATM-KO1 neurons. Genes found differentially expressed in A-T1 and ATM-KO1 relative to WT1 are listed in Suppl. Table 5. Specifically, in A-T1 neurons the number of up- and down-regulated genes accounted for 392 and 345, respectively, whereas in ATM-KO1 neurons 261 genes were down-regulated and only 24 up-regulated. A-T1 and ATM-KO1 neurons shared 2 genes that were upregulated and 26 down-regulated (Suppl. Fig. 10a,b). A Gene Ontology (GO) analysis performed on the downregulated gene set revealed an enrichment in biological processes related to neural development, maintenance and physiology of the CNS, a finding of potential relevance for the neuropathological abnormalities affecting the cerebellum, cerebrum, spinal cord and brain stem of A-T patients^2^. The heatmap of the genes annotated to the selected GO term (FN1, DCN, RASGRF1, FZD1, EOMES, SHH, NR2E1) is shown in Fig. 8b. The function of these genes is detailed in the Discussion section below and the legend to Suppl. Fig. 10.

## Discussion

A-T is an inherited multisystemic disorder with early onset brain degeneration, caused by mutations inactivating the ATM kinase best known for its apical role in DDR signalling. In accordance with this, neurodegeneration in A-T has been attributed to a dysfunctional DNA repair and genome instability accumulating during neurodevelopment, ultimately compromising the mature neurons. However, separate from its canonical role in DDR, ATM has been found involved in intracellular ROS sensing, protection from oxidative DNA damage and regulation of oxidative metabolism^4^, in ROS-dependent peroxisomal homeostasis via pexophagy^6^, and mitochondrial homeostasis through its capacity to regulate mitophagy^5^. The protection afforded by ATM against oxidative stress may be crucial for viability of neurons, which have an extremely high metabolic rate coupled with excessive ROS by-products. The loss of an effective antioxidant defence, as in ATM deficiency, may thus represent a contributing factor to neurodegeneration in A-T.

Neurodegeneration has also been linked to transcriptional decline and downregulation of synaptic genes in the CNS^10,11^. Moreover, the transcription of especially long genes depends on active Top1ccs, which introduce SSBs that reduce DNA torsion generated by elongating RNA pol II. The transiently formed Top1ccs are then removed to avoid conversion of SSBs into lethal DSBs^14^, and this process requires ATM, and indeed ATM deficient cells show an accumulation of Top1ccs as well as of aberrant DNA lesions and delayed transcriptional recovery^15–17^.

Here, we have shown that Top1 inhibition by CPT elicits in WT neurons an ATM-dependent signalling response that is absent in A-T neurons. Moreover, CPT induced transcriptional changes, including the upregulation of genes enriched for “cellular response to stress and hormones” and “RNA metabolism” and downregulation of genes enriched for “neural development and differentiation” “metabotropic signal transduction”, “nucleotides and metabolism”. In agreement with data showing that Top1 blocking impairs transcriptional elongation especially of long genes^23,25,26^, we found that the length of the CPT-downregulated transcripts was on average greater than those being upregulated in WT neurons. Notably, ATM inhibition, despite repressing the DDR, did not affect the length of the CPT-modulated genes, excluding ATM as having a role in Top1cc-dependent transcriptional elongation.

To investigate this issue more thoroughly, we performed RNA-seq analysis on nascent RNA from WT and A-T neurons exposed to CPT for 1 hr and then released in drug-free medium for 15 and 30 min to recover transcription. The bioinformatics analysis of the RNA-seq data performed both at transcript and exon level, however, did not show any differential transcriptional rescue behavior between WT and A-T neurons. Moreover, more detailed coverage analysis performed on transcripts subdivided into three length groups showed again no major differences in the efficiency of transcription recovery, altogether excluding a role for ATM in transcription elongation in postmitotic neurons.

Among the CPT-upregulated genes were those categorized as IEGs, which in the CNS are rapidly induced by synaptic activity and crucial for synaptic plasticity, learning and memory^28^. Of note, the induction of IEGs by CPT, seen at mRNA and protein level (for FOS and ARC), was independent of ATM protein expression or kinase activity.

Synaptic activity in mouse neurons induces the phosphorylation of proteins at S/TQ putative consensus sites for ATM/ATR kinases^31^. We have expanded these findings by demonstrating that human neuronal activity elicited by ionotropic glutamate receptors stimulation, or depolarization by KCl, causes the phosphorylation of different proteins at S/TQ sites, a phenomenon dependent on ATM as it is repressed in A-T and ATM-KO neurons. The lack of these phosphorylations in ATM-deficient neurons prompted us to determine the effects on IEGs, but regardless of the presence or activity of ATM, neuronal stimulation invariably induced the expression of IEGs, both at mRNA and protein level. These findings, coupled with the evidence that physiological stimuli in mouse neurons induce DSBs on IEGs promoters, necessary for their transcription^32,33^, suggest that the increased expression of γH2AX (a phosphorylation target of ATM and surrogate marker for DSBs) in our neurons is the consequence of activity-induced DSBs, as in mouse neurons. However, it remains to be established by a direct approach to which loci within the genome the potential DSBs map in our stimulus-responsive WT and ATM-deficient neurons.

To identify possible differences in activity-dependent gene expression in relation to ATM status, we performed RNA-Seq analysis in WT1, A-T1 and ATM-KO1 neurons depolarized with KCl, and for each cell type we identified a set of differentially expressed genes (up- and down-regulated) associated with neuronal activity. Despite the small number of DEGs identified, likely due to the short treatment time (1 hr), nevertheless the IEGs (eg: NR4A3, FOS, NR4A1 and NPAS4) were among those being upregulated regardless of ATM activity, again confirming the previous qRT-PCR and Western blot data.

We also sought to determine the impact of ATM deficiency on the transcriptional signature of unperturbed neurons and identified a set of genes (FN1, DCN, RASGRF1, FZD1, EOMES, SHH, NR2E1) that were downregulated in both A-T1 and ATM-KO1 compared to WT1. Of these, RASGRF1 is a Ca^2+^ activated protein involved in spinogenesis of primary hippocampal cultures. The nuclear receptor NR2E1 (also known as TLX) participates in synaptic plasticity and dendritic structure formation in the dentate gyrus^39^, in neurogenesis, learning and memory^40^. EOMES transcription factor regulates neurogenesis in the subventricular zone (SVZ)^41^. The morphogenic factor SHH is involved in the cerebellar development and proliferation of granular cell precursors, differentiation of Bergmann glial cells and normal Purkinje neuron development^42,43^.

In conclusion, we have shown that hiPSC-derived A-T neurons have an impaired DDR to CPT, like what seen with other genotoxic agents^16^. Additionally, CPT induces an ATM-independent expression of IEGs implicated in neuronal activity. We also established through nascent RNA-Seq analysis, that ATM deficiency does not affect transcription elongation, not even of long genes.

Most importantly, neuronal activity elicited by NMDA or AMPA receptor agonists, or depolarization with KCl, triggered an ATM-dependent DDR and an ATM-independent expression of IEGs. The ATM-dependent signalling might presumably reflects a response to the actual DSBs generated within promoters of IEGs^33^. In stimulated A-T neurons, albeit this signalling is repressed owing to ATM deficiency, the generation of DNA breaks is assumed to occur normally, as the induction of IEGs is not prevented. The role, if any, of ATM in the proper repair of these transient breaks is unknown. Finally, the transcriptomic analysis has revealed a set of genes whose basal downregulation in ATM-deficient neurons relative to WT neurons might be relevant for the neurodegenerative phenotype of A-T.

## Methods

An expanded version of the experimental procedures appears in the Supplementary data section that accompanies this manuscript.

### Fibroblast reprogramming and hiPSCs derivation

Human dermal fibroblasts were from a male healthy donor (WT1) (Coriell Institute Biobank, Camden, New Jersey, USA) and from an Ataxia Telangiectasia patient (A-T1). The A-T patient’s parents gave informed consent for skin biopsies and the Ethics Committee at the Children’s Memorial Health Institute (IPCZD) approved the study. Further, all methods were performed in accordance with the experimental protocols approved by the Ethics Committee at the Children’s Memorial Health Institute (IPCZD). Fibroblasts were cultured in DMEM F-12 supplemented with 15% Fetal Bovine Serum (both from Thermo Fisher Scientific), 2 mM L-glutamine, 0.1 mM non-essential aminoacids,100 units/mL penicillin, 100 µg/mL streptomycin (all from Lonza) and split with Trypsin/EDTA solution (LONZA). Integration-free hiPSCs clones were generated^44^ by transfecting 7 × 10^5^ fibroblasts with 3 μg of the episomal plasmids pCXLE-hUL (expressing L-MYC and LIN28; Addgene #27080), pCXLE-hOCT3/4-shp53-F (expressing OCT3/4 and shRNA against p53; Addgene #27077), pCXLE-hSK(expressing SOX2 and KLF4; Addgene #27078), and pAD-KLF4 (Addgene #19770) for the expression of the large isoform of Klf4 known to increase reprogramming efficiency^45^. Transfections were carried out using the Nucleofector solution Kit for Human Dermal Fibroblast and the Nucleofector I Device (U-20 program, Amaxa Biosystems, Lonza). Transfected fibroblasts were plated at a density of 35 × 10^3^ cells/well in a six well plate pre-coated with Matrigel hESC qualified, LDEV free (Corning) and cultured in medium without antibiotics for the first two days, and then in TeSR-E7 one (StemCell Technologies) until the appearance of iPSCs colonies. iPSCs clones were isolated from distinct colonies by manual picking after 22–26 days, and subsequently cultured using Matrigel coating and the mTESR1 medium (StemCell Technologies). The hiPSCs colonies were split using 0.5 mM EDTA in PBS. 1% RevitaCell Supplement 100 X (Thermo Fisher Scientific) was added after each passage to fresh mTESR1 medium overnight, to increase cell recovery. hiPSCs were cryopreserved in a solution containing 90% Knock Out Serum Replacement (KSR) (Thermo Fisher Scientific) and 10% DMSO (Sigma-Aldrich).

### Nascent mRNA isolation, TruSeq RNA Access library preparation, sequencing and bioinformatics analysis

Total RNA was purified from biological triplicates of treated and untreated neurons as described in the previous section and subjected to two rounds of oligo(dT) affinity capture using the Dynabeads mRNA purification Kit (Thermo Fisher Scientific) to recover the unbound poly(A)-minus fraction which is enriched for nascent RNA^46^. The poly(A)-minus fraction was then analysed by RT-PCR with RPPH1 and GAPDH probes to make sure that the depletion had been effective. For each samples, 10 ng of the poly(A)-minus RNA was used for library preparation using the TruSeq RNA Access library preparation kit (Illumina), which enriches for reads mapping to exons and allows a comprehensive coverage of coding RNA. Then, libraries were sequenced through a NextSeq 500 platform (Illumina) employing the 2 × 75 base pair (bp), paired end, HIGH flow cell sequencing (60 million reads/sample). Each experimental condition was tested as biological triplicate.

The bioinformatic analysis of the RNA-Seq raw dataset was carried our as follows. Briefly, counts generation was performed at both transcript-level and exon-level. Transcript-level reads were mapped to the reference human genome (hg19) with STAR v2.5.0, in stranded and pair-ends mode. Transcripts quantification was done with RSEM v 1.3.0 using GRCh37.75 ENSEMBL annotation. Exon-level reads mapping to the human genome (hg19) was done with bowtie v1.0.0, in stranded and pair-ends mode. Exons quantification was done using HTseq. 0.6.0 using GRCh37.75 ENSEMBL annotation after reorganizing exons features as requested by DEXSeq v1.12.2 (R 3.3.0). For differential expression analysis of transcripts we used DESeq2 1.14.1 (|log_2_FC| ≥1 and adjusted p-value ≤ 0.1) while for exons we used DEXSeq v 1.20.2 (|log_2_FC| ≥1 and adjusted p-value ≤ 0.1). Hierarchical clustering analysis was done with TMEV v4.8.1 using as clustering parameters Euclidean distance and average linkage. Oligonucleotide primers for PCR assay were for RPPH1 (forward: 5′GTCTGAGACTAGGGCCAGAG; reverse: 5′CATCTCCTGCCCAGTCTGAC) and for GAPDH (forward 5′TCACCAGGGCTGCTTTTAAC; reverse 5′TGGAAGATGGTGATGGGATT).

### RNA sequencing of KCl-depolarized neurons

Total RNA was purified from biological triplicates of treated and untreated neurons as described in the Real-Time quantitative PCR (qRT-PCR) section. Next generation sequencing experiments, comprising samples quality control and bioinformatics analysis, were performed by Genomix4life (Salerno, Italy). From each sample, indexed libraries were prepared from 50 ng of purified RNA with TruSeq RNA Access Sample Prep Kit (Illumina) according to the manufacturer’s instructions. Libraries were quantified using the TapeStation 4200 (Agilent Technologies) and Invitrogen Qubit fluorometer (Thermo Fisher Scientific), then pooled such that each index-tagged sample was present in equimolar amounts, with final concentration of the pooled samples of 2 nM. The pooled samples were subject to cluster generation and sequencing using an Illumina NextSeq 500 System (Illumina) in a 2 × 100 paired-end format at a final concentration of 1.8 pmol. Fastq underwent to Quality Control using FastQC tool (http://www.bioinformatics.babraham.ac.uk/projects/fastqc/). To remove the adapter sequences, cutadapt^47^ was used. The mapping of paired-end reads was performed using STAR (version 2.5.1a)^48^ on reference genome assembly HG38 obtained from Gencode v24^49^. The quantification of expressed transcripts for each of the sequenced biological replicates was performed using Cuffnorm (http://cole-trapnell-lab.github.io/cufflinks/cuffnorm/) algorithm of the Cufflinks program^50^. Cuffdiff (http://cole-trapnell-lab.github.io/cufflinks/cuffdiff/index.html) was used to perform the differential expression analysis. Differentially expressed genes were considered with cut-off of FDR ≤ 0.05 and Fold Change ≤ −1.5 for down genes and Fold Change ≥ 1.5 fo Up Genes. Gene Ontology Analysis (Biological Process; GO Ontology database Released 2017-10-24) was carried out using PANTHER (Protein ANalysis THrough Evolutionary Relationships) Classification System Version 12.0 (released 2017-07-10)^51^.

### Use of human samples

For iPSC reprogramming, human dermal fibroblasts from normal donors (WT1 and WT2) and from an A-T patient (A-T2) were obtained from the Coriell Institute Biobank (Camden, New Jersey, USA). Dermal fibroblasts for the sample labelled A-T1 were obtained from an A-T case for which an informed consent and Ethics committee approval was obtained at the The Children’s Memorial Health Institute (IPCZD), Warsaw.

## Supplementary information

Supplementary information

Dataset 1

Dataset 2

Dataset 3

Dataset 4

Dataset 5

## Data Availability

The Exon Microarray data reported in this paper have been deposited in the Gene Expression Omnibus under GEO: GSE108605. Additional data generated during the current study are available from the corresponding author on request.
