# Supplementary material for: DNA damage and transcriptional regulation in iPSC-derived neurons from Ataxia Telangiectasia patients
Source: Sci Rep. 2019 Jan 24;9:651. doi: 10.1038/s41598-018-36912-0 (PMC6346060; doi:10.1038/s41598-018-36912-0)
Supplement: Supplementary file 1 — Supplementary information [file 41598_2018_36912_MOESM1_ESM.pdf]

DNA damage and transcriptional regulation in iPSC-derived neurons from Ataxia Telangiectasia patients.

Alessandro Corti, Raina Sota, Matteo Dugo, Raffaele A. Calogero, Benedetta Terragni, Massimo Mantegazza, Silvana Franceschetti, Michela Restelli, Patrizia Gasparini, Daniele Lecis, Krystyna H Chrzanowska and Domenico Delia

Supplementary information

Legends to Figures

Suppl Fig 1: Characterization of the WT1 and A-T1 hiPSCs.

hiPSCs were generated as reported in Materials&Methods. **A,** The clones shown here, used for all subsequent experiments, were checked by IF for the expression of Oct3/4, SSEA4 and Tra1-81 pluripotency markers. **B,** Differentiation of these hiPSCs into the three germ layers expressing markers for the ectoderm (β-Tub III)), endoderm (Sox17) and mesoderm (α-SMA). **C,** Cytogenetic analysis of WT1 and A-T1 shows karyotypes without evident chromosomal abnormalities. **D**, Western blot analysis of the primary fibroblasts and their hiPSC derivatives, showing expression of the pluripotency marker Oct3/4 by the latter. Vinculin and β-Actin are protein loading controls.

Suppl Fig 2: Characterization of the hiPSC-derived neural progenitor cells (hNPCs) before and after differentiation into mature neurons.

A, Expression by hNPCs of the precursor markers Nestin and SOX2. **B,** Upon differentiation, most cells become positive for MAP2, a marker of mature neurons, and few for the astrocytic marker GFAP. **C,** Western blot analysis carried out at various times after *in vitro* differentiation of hNPCs, shows the loss of the proliferation marker Ki67 and of Nestin (left panel), and a reciprocal rise of the β-Tubulin III and MAP2 neuronal markers, as well as expression of GFAP. In the right panel the time-dependent expression of neuronal maturation markers PSD5, Synaptosin, SCG10 and pan-KCHIP is evident. β-Actin and vinculin used as a protein loading controls. It should be noted that, especially at DIV55, GFAP expression is markedly higher in A-T1 than WT1 neuronal cultures.

Suppl Fig 3: Expression of excitatory and inhibitory genes by WT and AT mature neurons.

The histogram reports the log2 fold levels of the indicated transcripts as determined by exon microarray analysis performed on DIV55 WT and A-T neurons. It can be seen that the neurons express cortical excitatory and inhibitory markers.

Suppl Fig 4: Electrophysiological recordings from WT1 and A-T1 neurons.

Recordings were performed on DIV55 neurons. **A**, 96% of WT1 and 100% of A-T1 neurons showed the expression of voltage-gated sodium and potassium currents, the main ionic conductances characterizing mature neurons. Peak sodium current densities and potassium current densities measured at +20 mV were similar in both types of neurons (INa WT1: 69.8±6.9 pA/pF, n=20, INa A-T1: 67.8±610.3 pA/pF, n=12, t-test, p>0.05; IK WT1: 48.5±6.0 pA/pF, n=20, IK A-T1: 35.6±2.3 pA/pF, n=12, t-test, p>0.05). **B**, spontaneous postsynaptic currents recorded at -50 mV (sEPSCs) or 0 mV (sIPSCs) from representative WT1 (top) and A-T1 (bottom) neurons (scale bars: 15 pA/10 s). **C**, WT1 (top) and A-T1 (bottom) discharges of action potentials evoked by the injection of 2.5 s-long depolarizing current pulses of increasing amplitude from a resting potential of -70 mV (scale bars: 10 mV/0.5 s).

Suppl Fig 5: CPT-induced nuclear foci and phosphorylation of ATM substrates.

A, CPT-treated DIV30 neurons were IF labelled to visualize DNA damage-associated 53BP1 nuclear foci. The genotoxic agent NCS (neocarzinostatin) was used as positive control for foci formation. **B**, the Western blots were performed on lysates from DIV30 neurons that were pre-treated (or not) with Kudos55933 to inhibit ATM (ATMi). β-Actin and Vinculin served as protein loading controls.

Suppl Fig 6: AMPA-stimulated neurons activate the DDR.

DIV55 WT2 neurons were pre-incubated overnight with TTX and then treated with AMPA for up to 30 min. The Western blot shows the AMPA-induced phosphorylation of ATM, Kap1 and Chk2 especially at 20min. β-Actin is a protein loading control.

Suppl Fig 7: Generation and characterization of ATM knockout hNPCs by CRISPR system.

The parental WT1 hNPCs were employed to knockout the ATM gene using the CRISPR-GFP system, as detailed in Materials and Methods. **A**, The resulting ATM-KO1 hNPCs are mostly negative by IF staining for ATM, in contrast to the parental cells being all positive. The loss of ATM, however, does not affect the expression of the neural precursor markers Nestin and Sox2. **B**, Western blot showing a strong reduction of ATM levels in ATM-KO1 cells compared to the parental hNPCs, a finding in accordance with the IF data. **C**, Double IF performed on DIV20 neurons labelled with antibodies against MAP2 (green) and ATM (red), showing negative staining for ATM by the majority of neurons derived from the ATM-KO1 hNPCs. **D**, Western blot showing loss of the proliferation marker Cyclin-B1 and reciprocal induction of β-Tubulin III upon hNPCs differentiation. β-Actin served as protein loading control.

Suppl Fig 8: Venn diagram of activity-dependent differentially expressed genes in WT1, A-T1 and ATM-KO1 neurons.

DIV55 neurons were treated with KCl for 1hr and analysed by RNAseq. The numbers indicate the up- and down-regulated differentially expressed genes that are unique to each cell type or common between them. Specifically, a small set of genes that included NR4A3, FOS, NR4A1 and NPAS4, was strongly up-regulated after treatment in all three cell lines.

Suppl Fig 9: qRT-PCR analysis of FOS

Neurons at DIV55 were treated with KCl for 1hr and then analysed by qRT-PCR with primers specific for FOS and GAPDH (for normalization). Results are from two independent experiments and values +/-SD for FOS were normalised against those of GAPDH (** $p<0.01$).

Suppl Fig 10: Venn diagram of basal differentially expressed genes in WT1 compared to A-T1 and ATM-KO1 neurons.

Transcripts that are differentially expressed between WT1, A-T1 and ATM-KO1, identified by RNA-seq analysis of unstimulated neurons. Numbers indicate the differentially expressed genes that are unique or common between each group.

FN1, DCN, RASGRF1, FZD1, EOMES, SHH, NR2E1 showed an ATM-dependence, being expressed in WT1 but not in A-T1 or ATM-KO1. Of these genes, RASGRF1, a

Ca^{2+} activated protein, is involved in spinogenesis of primary hippocampal cultures, while the nuclear receptor NR2E1 (also known as TLX) participates in synaptic plasticity and dendritic structure formation in the dentate gyrus [47] (Christie et al, 2006), as well as neurogenesis, learning and memory [48] (Murai et al, 2014). Moreover, lower expression levels were detected in A-T1 and ATM-KO1 neurons for the T-box transcription factor EOMES, a key regulator of neurogenesis in the subventricular zone (SVZ) [49] (Arnold et al, 2008) and for the morphogenic factor SHH. Down-regulation of SHH could be particularly interesting since this gene is involved in cerebellum in the proliferation of granular cell precursors, differentiation of Bergmann glial cells and normal Purkinje neuron development [50, 51] (De Luca et al, 2016; Lee et al, 2010). Curiously, we noticed another A-T1 and ATM-KO1 common down-regulated GO Term, described as "negative regulation of cell death" (GO:0060548), in which we found the following genes: SHH, CTGF, NR2E1, CARD16. This could suggest that the absence of ATM might make neurons more apoptogenic. In addition to the shared GO terms, we focused on two categories associated with DDR induced by ionizing radiation, a finding in accordance with the marked radiosensitivity of A-T cells. Specifically, A-T1 neurons displayed two down-regulated genes belonging to the cellular response to X-ray (GO:0071481) while ATM-KO1 neurons had lower expression levels for three genes associated with cellular response to γ-radiation (GO:0071480) (data not shown).

Additional Experimental procedures:

Karyotype analysis. hiPSCs were incubated overnight with 20ng/ml of the mitotic inhibitor Colcemid (Thermo Fisher Scientific), thereafter incubated in hypotonic solution (KCl; 1 h at 37°C), and then fixed with Carnoy's fixative (3:1 methanol to acetic acid). Metaphase spreads were aged at room temperature for 5-7 days and banded with Wright stain.

Embryoid bodies generation and differentiation into cells of the 3 germ-layers. hiPSCs colonies were incubated for 10 min at 37°C with 1mg/ml Collagenase type IV (Thermo Fisher Scientific) dissolved in DMEM F-12. After scraping, heterogeneous cell clumps were transferred into ultralow attachment six-well plates (Euroclone) and kept in a medium optimized for EBs growth (DMEM F-12, 20% KSR, 2 mM L-glutamine, 0.1 mM non essential aminoacids,100 units/mL penicillin, 100 µg/mL streptomycin, 1mM Sodium Pyruvate from LONZA, 1% N-2 Supplement (Thermo Fisher Scientific), 0.11mM β-ME (Sigma Aldrich). After 6 days of floating cell culture, EBs were transferred onto coverslips (13mm diameter) pre-coated with Geltrex^{TM} LDEV Free Reduced Growth Factor Basement Membrane Matrix (Thermo Fisher Scientific) and kept for 7 days in a 3 germ layer differentiation medium (DMEM F-12, FBS 20%, 2 mM L-glutamine, 0.1 mM non essential aminoacids ,100 units/mL penicillin, 100 µg/mL streptomycin). Medium changes were performed 3 times per week for both floating and adherent cell cultures. At the end of the differentiation step, coverslips were fixed and immunostained with antibodies against the three germ layers (see Immunofluorescence section).

Generation of neural precursor cells (hNPCs) from hiPSCs. Proliferating neural precursor cells (NPCs) were obtained as reported (1S). Essentially, hiPSCs clones were, the day before neural induction, split to get a 20% confluent cell culture; after that mTESR1 medium was replaced with the PSC Neural Induction Medium containing Neurobasal medium, 2% Neural Induction Supplement 50X (all from Thermo Fisher Scientific), 100 units/mL penicillin, and 100 µg/mL streptomycin, and cells cultured for 7 days. Induced cells were subsequently dissociated with Accutase solution (Carlo Erba Reagents), counted and seeded on a Geltrex pre-coated dish at $1x10^5$ cells/cm^2 in the Neural Expansion Medium (50% Neurobasal medium, 50% Advanced DMEM F-12, 2% Neural Induction Supplement 50X, 100 units/mL

penicillin, 100 μg/mL streptomycin). The medium was supplemented overnight with 1% RevitaCell Supplement, to improve cell viability. After induction, hNPCs were split with Accutase solution and used for terminal differentiation for no longer than 25 passages. During initial passages, the hNPCs were selectively isolated from the cultures containing non-neural cells through a differential gentle detachment with Accutase as recommended (Induction of Neural Stem Cells from Human Pluripotent Stem Cells Using PSC Neural Induction Medium, Appendix B, protocol published by Thermo Fisher Scientific). The hNPCs were cryopreserved at different passages in Neural Expansion medium supplemented with 10% DMSO.

ATM gene knockout by CRISPR/Cas9. To knockout the ATM gene, we used the CRISPR/Cas9 plasmid pSpCas9(BB)-2A-GFP (PX458) (Addgene #48138) [2S] expressing two sgRNAs selected using a standard in silico prediction tool, one targeting exon 3 and one exon 6 of the ATM gene. WT1 hNPCs were transfected with the plasmids using the Nucleofector Kit solution in combination with the Amaxa nucleofector set on the A-33 program. After 24 hrs, GFP-positive NPCs were purified with the FACS cell sorter to enrich for the ATM knockout fraction, and subsequently expanded. ATM ablation in the hNPCs was verified by Western blot and IF analysis. Guide RNA sequences targeting ATM exons are: 5'TTGTTTCAGGATCTCGAATC (Exon 3) and 5'TTTTGGCTCCTTTCGGATGA (Exon 6).

Terminal neural differentiation. After six passages, hNPCs were seeded at a $5x10^4$ $cells/cm^2$ in a 2 fold concentrated Geltrex pre-coated plate and cultured with a neural differentiation medium (NDM) containing: Neurobasal medium, 2% B27 supplement-serum free, 2mM GlutaMAX supplement (Thermo Fisher Scientific), 10ng/μl brain-derived neurotrophic factor, 10ng/μl glial cell-derived neurotrophic factor (both from Immunotools), 200μM L-ascorbic Acid (Merck), 0.1 mM non essential aminoacids, 100 units/mL penicillin, 100 μg/mL streptomycin. During the differentiation, which was carried out for up to 55 days *in vitro* (DIV), 75% of the spent NDM was replaced with fresh NDM three times/week.

Drug treatments. Neurons were treated with 30μM camptothecin (CPT, Sigma Aldrich) for 1 hour, then washed with PBS and replenished with drug-free pre-conditioned medium and cultured for 3hrs. Neocarzinostatin (NCS, Sigma Aldrich)

was used at 3.32 nM for 20 min as positive control for DSBs induction. For stimulation studies, DIV55 neurons were incubated overnight with 2µM tetrodotoxin (TTX, from Tocris Bioscience) to silence activity, then treated with either 50mM potassium chloride (KCl) to induce membrane depolarization, or 50µM *N*-Methyl-D-aspartic acid (NMDA), or 50µM α-amino-3-hydroxy-5-methyl-4-isoxazolepropionic acid (AMPA), all purchased from Sigma-Aldrich. The AMPA potentiator S70340, used at 20µM, was a gift of Pierre Francotte Laboratory, University of Liege, Belgium To inhibit ATM kinase, neurons were pre-incubated for 1hr with 10µM KU55933 (Sigma Aldrich) before other treatments. The NMDA receptor antagonist MK801 (Tocris Bioscience) was added (20µM) 1 hr prior treatment with NMDA.

Electrophysiological recordings. Whole-cell patch-clamp recordings were performed as described [29, 3S] on DIV55 neurons at room temperature (~25 °C) using a Multiclamp 700A amplifier and pClamp 10.5 software (Molecular Devices, Sunnyvale, CA, USA) in voltage- or current-clamp configuration. External bath solution contained (mM): 129 NaCl, 1.25 NaH_2PO_4, 35 glucose, 1.8 $MgSO_4$, 1.6 $CaCl_2$, 3 KCl and 10 HEPES, pH 7.4 with NaOH. The internal pipette solution contained (in mM): 120 K-gluconate, 15 KCl, 2 $MgCl_2$, 0.2 EGTA, 10 HEPES, 20 phosphocreatine-tris, 2 ATP-Na_2, 0.2 mM GTP-Na_2 and 0.1 leupeptin, pH 7.2 with KOH. Pipette resistance was between 3 and 4 MΩ. Cell capacitance and series resistance errors were carefully compensated (~85%) throughout the experiment. The remaining linear capacity and leakage currents were eliminated online using a P/4 subtraction paradigm. Total voltage-gated currents were elicited by applying 66 ms-long depolarizing voltage steps from -70 to +50 mV (10 mV increments), from a holding potential of -70 mV; signals were filtered at 10 kHz and sampled at 200 kHz. Discharges of action potentials were evoked by the injection of 2.5 s-long depolarizing current pulses of increasing amplitude from the resting potential maintained at -70 mV; signals were filtered at 10 kHz and sampled at 20 kHz. Spontaneous excitatory and inhibitory postsynaptic currents (sEPSCs, sIPSCs) were recorded at -50 and 0 mV, respectively; signals were filtered at 3 kHz and sampled at 10 kHz.

Western blots and immunofluorescence. Western blot analysis were performed as reported [16] Briefly, cells detached with Accutase were washed and lysed in 20-

100μl of lysis buffer (0.125 M Tris-HCl pH 6.8, 5% SDS) containing proteases and phosphatases inhibitors. Following sonication, centrifugation and quantification samples were loaded (30-50μg) on Novex NuPAGE precast gels, electrophoresed and blotted on PVDF membranes (Merck). After blocking in 4% nonfat dry milk , membranes were incubated with primary antibodies overnight at 16°C using the X-BLOT P100 System (Isenet, Milano, Italy), then for 1hr with horseradish peroxidase-conjugated secondary antibodies (GE Healthcare). Proteins were detected by chemiluminescence and signals quantitated by densitometric analysis using the ImageQuant 5.2 software (Molecular Dynamics). Total protein loading per lane was normalized with antibodies against β-Actin and Vinculin. For immunofluorescence (IF), cells seeded onto 13 mm round coverslips were fixed for 10 mins with 4% paraformaldehyde (PFA, Sigma Aldrich), washed for 5 min with TBS (NaCl 0,9%, Tris HCl 50mM pH 7.4) and either kept in PBS at 4°C or stained immediately. For intracellular staining, fixed cells were permeabilized with 0.1% Triton X-100 (Sigma Aldrich) for 10 min and then blocked with 10% normal goat serum (NGS) for 30 min. Coverslips were incubated with primary antibodies diluted in 5% NGS for 3 hours at RT or overnight at 4°C, washed 3x with PBS and then incubated for 45 min at RT with appropriate dilutions of Alexa Fluor555 and 488 conjugated secondary antibodies (Thermo Fisher Scientific). Following washing with PBS, coverslips were incubated for 10 min with 0.35μg/ml of DAPI nuclear stain, mounted onto microscope slides using Prolong Gold antifade reagent (Thermo Fisher Scientific) and analyzed on a Nikon Eclipse E1000 fluorescence microscope equipped with a DS-U3 CCD digital camera. To increase the quality of Tra1-81 and SSEA4 IF staining, iPSCs dissociated into single cells were cytocentrifuged (500 rpm, 5 min) onto microscope glass slides with a Shandon Cytospin 2 Centrifuge (Block Scientific, Inc.), air-dried overnight and then fixed with PFA fixation.

Primary antibodies and dilutions used for WB and IF in this study:

Antibody	Identifier	Source	Dilution	WB	IF
ATM	#1549-1	Epitomics	1:1000		X
ATM	#ab32420	Abcam	1:1000	X	
ATM-pS1981	#13050	Cell Signaling Technology	1:1000	X	
KAP1	#5868	Cell Signaling Technology	1:1000	X	
KAP1-pS824	#4127	Cell Signaling Technology	1:1000	X	
KAP1-pS473	#644602	BioLegend	1:1000	X	
γ-H2AX	#A300-081A	Bethyl Laboratories Inc.	1:1000	X	
Chk2-pT68	#2661	Cell Signaling Technology	1:1000	X	
Chk2	#NBP1-97546<br>#NBP1-97549	Novus Biologicals	1:1000	X	
Chk2	#44D4-21	Generated in house	1:1000	X	
p53-pS15	#9284	Cell Signaling Technology	1:1000	X	
p53	#sc-47698	Santa Cruz Biotechnology	1:500	X	
Oct-3/4	#sc-5279	Santa Cruz Biotechnology	1:1000;<br>1:200	X	X
Tra1-81	#14-8883-82	Thermo Fisher Scientific	1:200		X
SSEA4	# MC-813-70	Thermo Fisher Scientific	1:10		X
Nanog	#ab21624	Abcam	1:500	X	
Sox2	#3579	Cell Signaling Technology	1:1000	X	
Sox2	#11064-1-AP	Proteintech Europe	1:50		X
c-Fos	#4384	Cell Signaling Technology	1:1000	X	
Cleaved-PARP Asp214	#9541	Cell Signaling Technology	1:1000	X	
β3-Tubulin, Class III (TUJ1)	#T8578	Sigma-Aldrich	1:10000	X	
β3-Tubulin, Class III (TUJ1)	#MMS-435P-250	BioLegend	1:5000		X
Ki-67	#M7240	Agilent Dako	1:1000	X	
MAP2	#MAB3418	Merck Millipore	1:5000	X	X
GFAP	#AB5804	Merck Millipore	1:4000		X
GFAP	#AB5804	Merck Millipore	1:2000	X	
Nestin	#MAB5326	Merck Millipore	1:1000	X	X
Synaptophysin	#61-614	NeuroMab	1:1000	X	
PSD95	#75-028	NeuroMab	1:1000	X	
Pan-KChIP	#75-006	NeuroMab	1:1000	X	
SCG10 (Stathmin-2)	#75-063	NeuroMab	1:1000	X	
VGluT1	#135303	Synaptic Systems	1:3000	X	
VGAT	#131011	Synaptic Systems	1:1000	X	
ARC	16290-1-AP	Proteintech Europe	1:1000	X	
α-SMA	#A2547	Sigma Aldrich	1:400		X
cyclin B1	#554176	BD Pharmigen	1:1000	X	
53BP1	#NB100-304	Novus Biologicals	1:750		X
β-Actin	#A1978	Sigma-Aldrich	1:20000	X	
Vinculin	#V9131	Sigma-Aldrich	1:5000	X	

53BP1 foci immunodetection.

Neurons grown on coverslips were treated with genotoxic agents, then fixed with 2% PFA for 20 min, washed and treated with a permeabilization solution (20mM HEPES buffer, 50mM NaCl, 3mM MgCl2, 300mM sucrose, 0.5%Triton X-100, pH 7.6) for 5 minutes. After blocking with 5% BSA (Sigma Aldrich) and 0.1% Tween-20 for 10min, coverslips were incubated with the 53BP1 antibody in 2% BSA for 45 min at RT, then extensively washed and incubated for 45min at RT with Alexa-Fluor555 secondary antibody diluted in 1% BSA. Following washing and counterstaining with DAPI, coverslips were mounted as above.

Exon microarray analysis

RNA was extracted from three independent experiments of untreated and treated neuronal cultures that were lysed in Qiazol reagent (Qiagen, CA, USA), and further purified with the RNeasy Mini Kit (Qiagen) according to the manufacturer's instructions. RNA quality and quantity were determined using the Bioanalyzer (Agilent, USA) and the Nanodrop spectrophotometer (Thermo Scientific). Total RNA was amplified with the Agilent Low Input Quick Amp WT labeling kit. Briefly, 100 ng of total RNA was used to synthesize double-stranded cDNA, amplified by *in vitro* transcription and labelled with cy3-dCTP. Fluorescent dye-labelled cRNA was hybridized on 2×400K Agilent Human SurePrint G3 exon microarray slides. Hybridization and washing were performed according to Agilent's standard protocols. Microarray images were acquired using an Agilent DNA microarray scanner and raw data were generated using the Feature Extraction Software v10.7.3 involving automatic grid positioning, intensity extraction (signal and background). Raw data were preprocessed using the limma package [4S]. Briefly, after background correction with the *normexp* method, raw data were normalized with quantile method and log2 transformed. Spots detected in at least two samples according to the *gIsPosAndSignif* and *gIsWellAboveBG* flags were kept for further analyses. Finally the dataset was collapsed at the gene level calculating the mean expression of probes mapping on the same gene. Data were deposited in the Gene Expression Omnibus repository (GSE108605). Differentially expressed genes were identified using the linear model approach implemented in the limma package. Multiple-testing correction was performed using the Benjamini-Hochberg false discovery rate (FDR) [4S] . Genes with FDR < 0.05 and absolute fold-change ≥ 2 were considered significant. Pre-

ranked Gene Set Enrichment Analysis (GSEA) [6S] was run on the C5 BP collection (Gene Ontology Biological Process terms) from the Molecular Signatures Database using the t-statistics from limma as ranking metric. GSEA results were visualized using the EnrichmentMap [7S] plugin for Cytoscape v3.5.1.

Real-Time quantitative PCR (qRT-PCR).

Neurons were dissociated with Accutase, centrifuged at 4500 rpm for 5 min, washed in PBS plus 0.5% BSA and 2mM EDTA (Thermo Fisher Scientific), pelleted and lysed in 500µl of Qiazol Lysis Reagent. Total RNA was then extracted and purified with the miRNeasy Mini Kit followed by treatment with RNase Free DNase Set and a second step of isolation with the RNeasy MinElute cleanup kit (all reagents from QIAGEN). The purified RNA was quantified with the Nanodrop 2000c spectrophotometer (Thermo Fisher Scientific) and 1µg was retro-transcribed using the Transcriptor First Strand cDNA Synthesis Kit (Roche) to get a 50µg/µl concentrated cDNA solution. Real Time PCR assays were performed on 20 ng of cDNA for each sample mixed with sterile water, Fast SYBR^{TM} Green Master Mix (Thermo Fisher Scientific) and the specific forward and reverse primer sets. Each sample was dispensed into three wells (technical triplicate) of the MicroAmp Fast Optical 96-well Reaction Plate with Barcode (0.1ml) and analyzed by a ViiA 7 Real-Time PCR System (all from Thermo Fisher Scientific). All results are expressed as fold changes (FCs), calculated with the $\Delta\Delta$Ct method, and normalized on GAPDH cDNA levels. The sequences of the primers (Eurofins Genomics) are:

Target Gene	Primer Forward (5'-3')	Primer Reverse (5'-3')
ARC	GCGGCTCTGAGGAGTACTGGCT	ATGGCCTCTCGGGACAGCGT
FOS	TTACTACCACTCACCCGCAG	AGTGACCGTGGGAATGAAGT
EGR1	GCCATAGGAGAGGAGGGTTC	GCAGAAAGGATTGTTGGCCA
GAPDH	AATCCCATCACCATCTTCCA	TGGACTCCACGACGTACTCA

Supplemental references

1S. Yan Y1, Shin S, Jha BS, Liu Q, Sheng J, Li F, Zhan M, Davis J, Bharti K, Zeng X. (2013). Efficient and rapid derivation of primitive neural stem cells and generation of brain subtype neurons from human pluripotent stem cells. Stem Cells Transl Med. 2013; 2:862-870.

2S. Ran FA, Hsu PD, Wright J, Agarwala V, Scott DA, Zhang F. Genome engineering using the CRISPR-Cas9 system. Nat Protocols. 2013. 8:2281-2308.

3S. Terragni B, Scalmani P, Colombo E, Franceschetti S, Mantegazza M. Ranolazine vs phenytoin: greater effect of ranolazine on the transient Na+ current than on the persistent Na+ current in central neurons. Neuropharmacology. 2016. 110: 223-236

4S. Ritchie ME, Phipson B, Wu D, Hu Y, Law CW, Shi W, Smyth GK. Limma powers differential expression analyses for RNA-sequencing and microarray studies. Nucleic Acids Res. 2015. 43, e47.

5S. Benjamini Y, Hochberg Y. Controlling the False Discovery Rate: A Practical and Powerful Approach to Multiple Testing. 1995. J. R. Stat. Soc.57, 289–300.

6S. Subramanian A, Tamayo P, Mootha VK, Mukherjee S, Ebert BL, Gillette MA, Paulovich A, Pomeroy SL, Golub TR, Lander ES, et al. Gene set enrichment analysis: a knowledge-based approach for interpreting genome-wide expression profiles. Proc Natl Acad Sci U S A. 2005. 102:15545-15550

7S. Merico D, Isserlin R, Stueker O, Emili A, Bader GD. Enrichment map: a network-based method for gene-set enrichment visualization and interpretation. PLoS One.2010. 5, e13984.

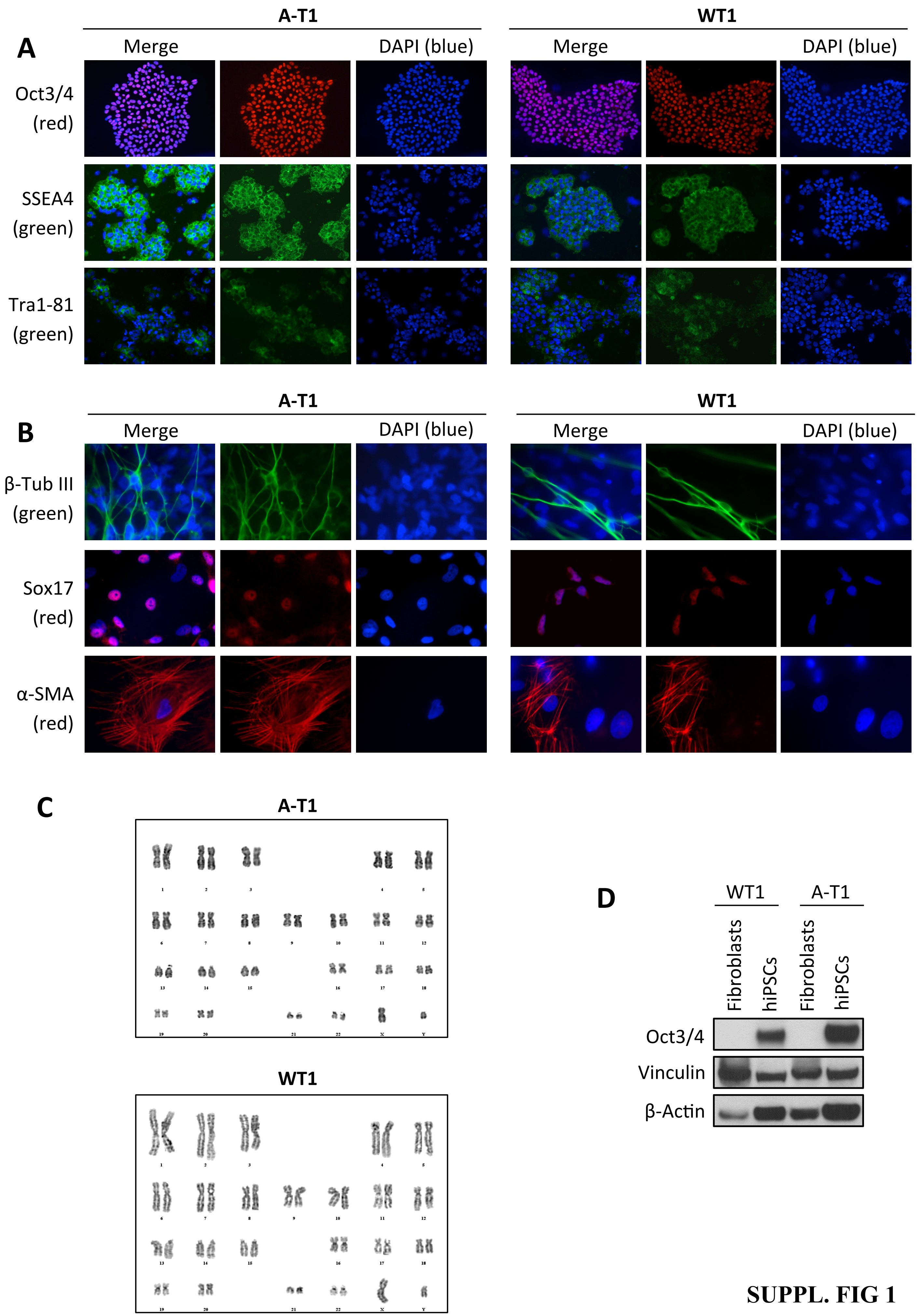
A
A-T1
WT1
Merge
DAPI (blue)
Merge
DAPI (blue)
Oct3/4 (red)
SSEA4 (green)
Tra1-81 (green)
B
A-T1
WT1
Merge
DAPI (blue)
Merge
DAPI (blue)
β-Tub III (green)
Sox17 (red)
α-SMA (red)
C
A-T1
WT1
D
WT1
A-T1
Fibroblasts
hiPSCs
Fibroblasts
hiPSCs
Oct3/4
Vinculin
β-Actin

SUPPL. FIG 1

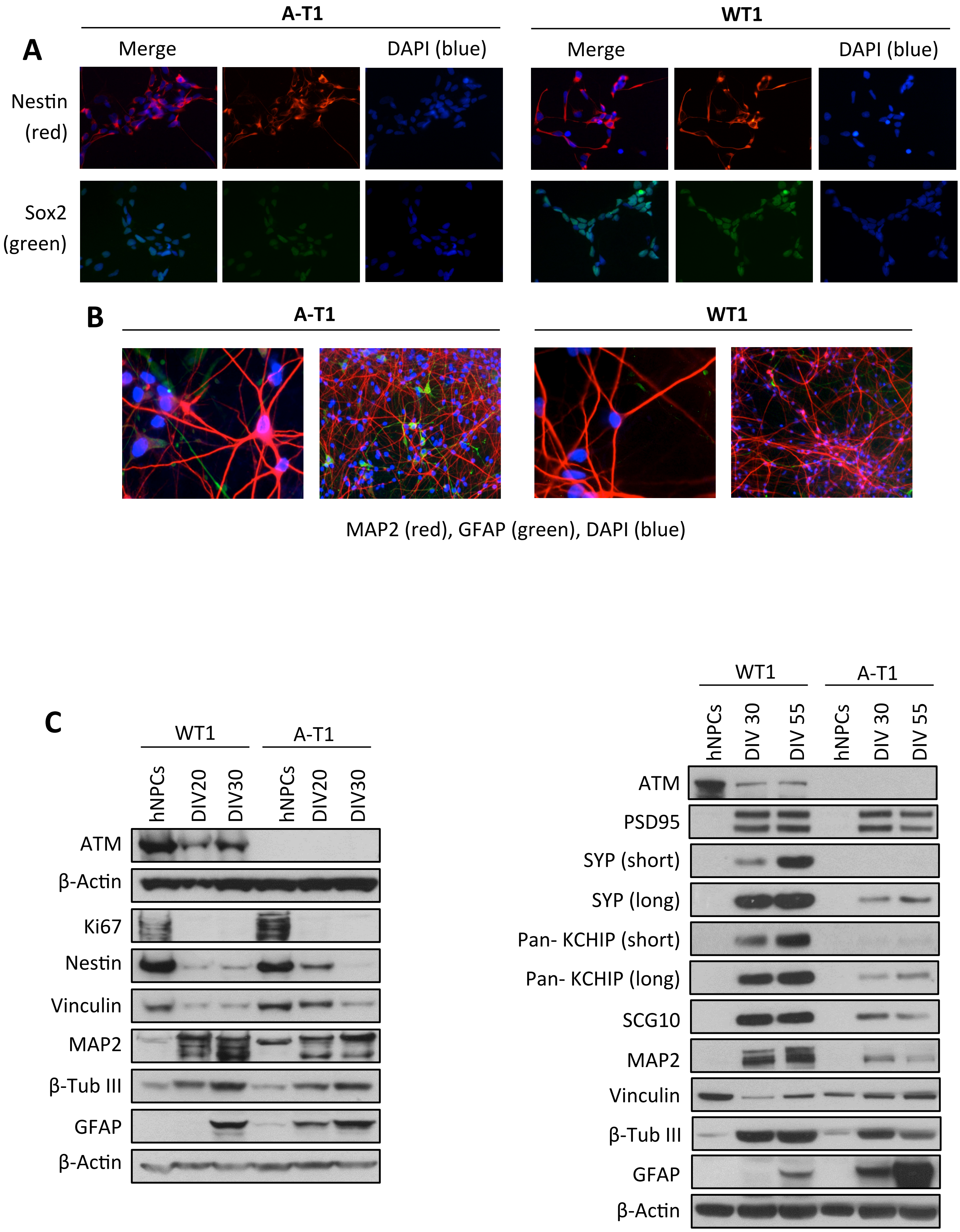
A
A-T1
WT1
Merge
DAPI (blue)
Nestin (red)
Sox2 (green)
B
MAP2 (red), GFAP (green), DAPI (blue)
C
hNPCs
DIV20
DIV30
ATM
β-Actin
Ki67
Nestin
Vinculin
MAP2
β-Tub III
GFAP
DIV 30
DIV 55
PSD95
SYP (short)
SYP (long)
Pan- KCHIP (short)
Pan- KCHIP (long)
SCG10

SUPPL. Fig 2

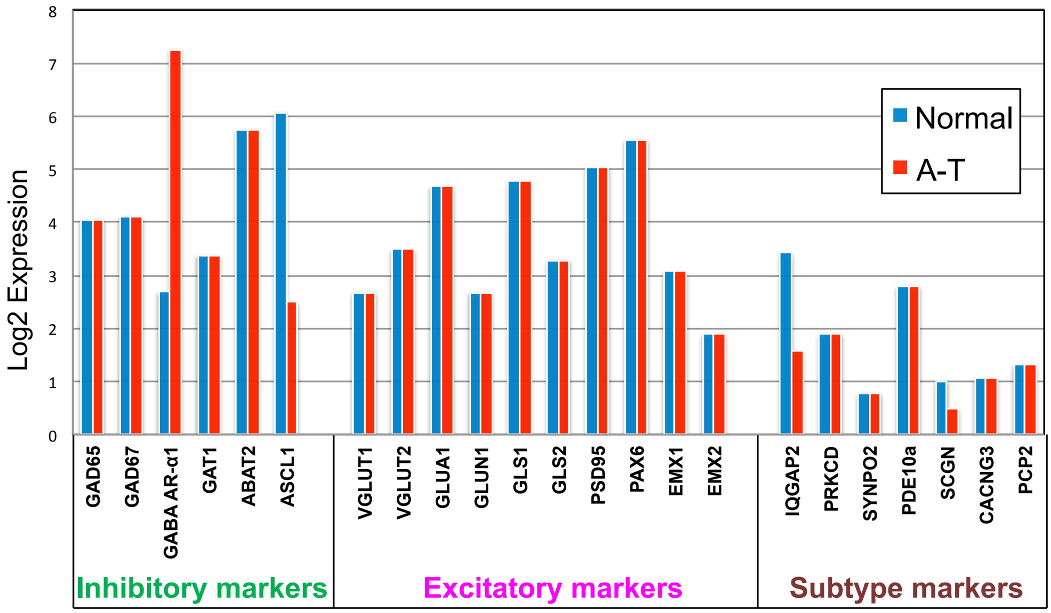

IQGAP2: Hippocampus
PRKCD: Thalamus
SYNPO2: Thalamus
PDE10A: Striatum
SCGN: Olfactory bulb
CACNG3: Cortex
PCP2: Cerebellum

SUPPL. Fig 3

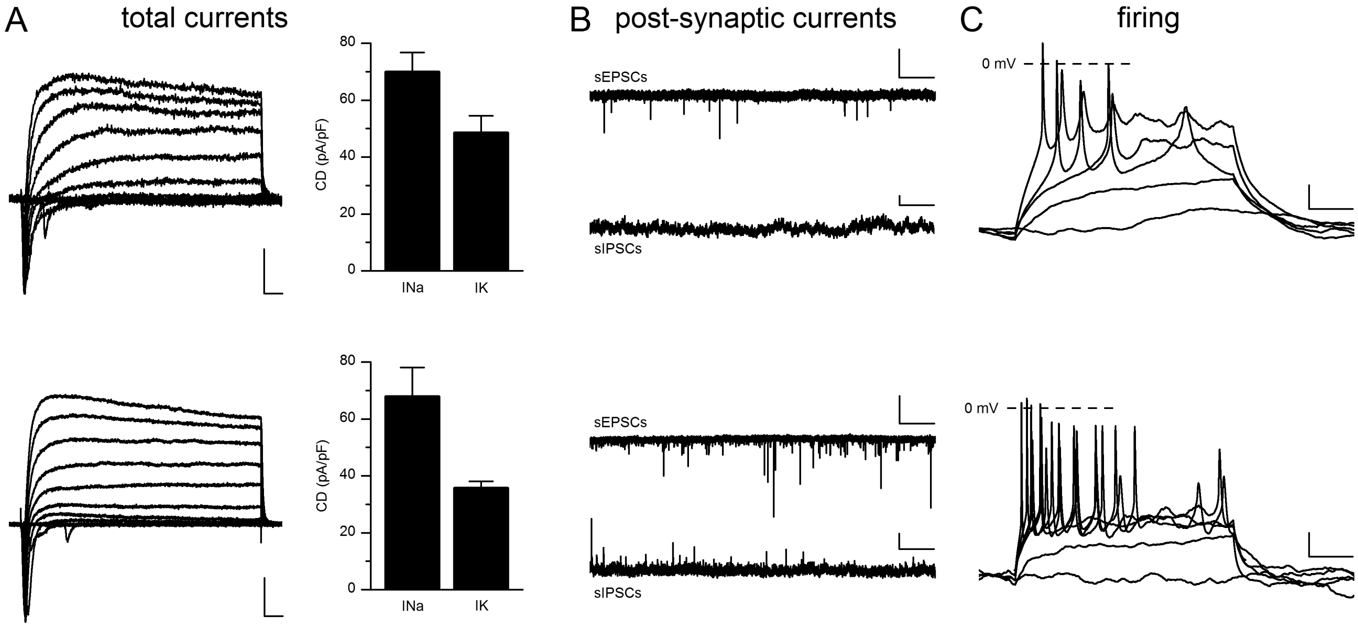
A
total currents
80
60
40
20
0
CD (pA/pF)
INa
IK
80
60
40
20
0
CD (pA/pF)
INa
IK
B
post-synaptic currents
sEPSCs
sIPSCs
sEPSCs
sIPSCs
C
firing
0 mV
0 mV

SUPPL. Fig 4

A

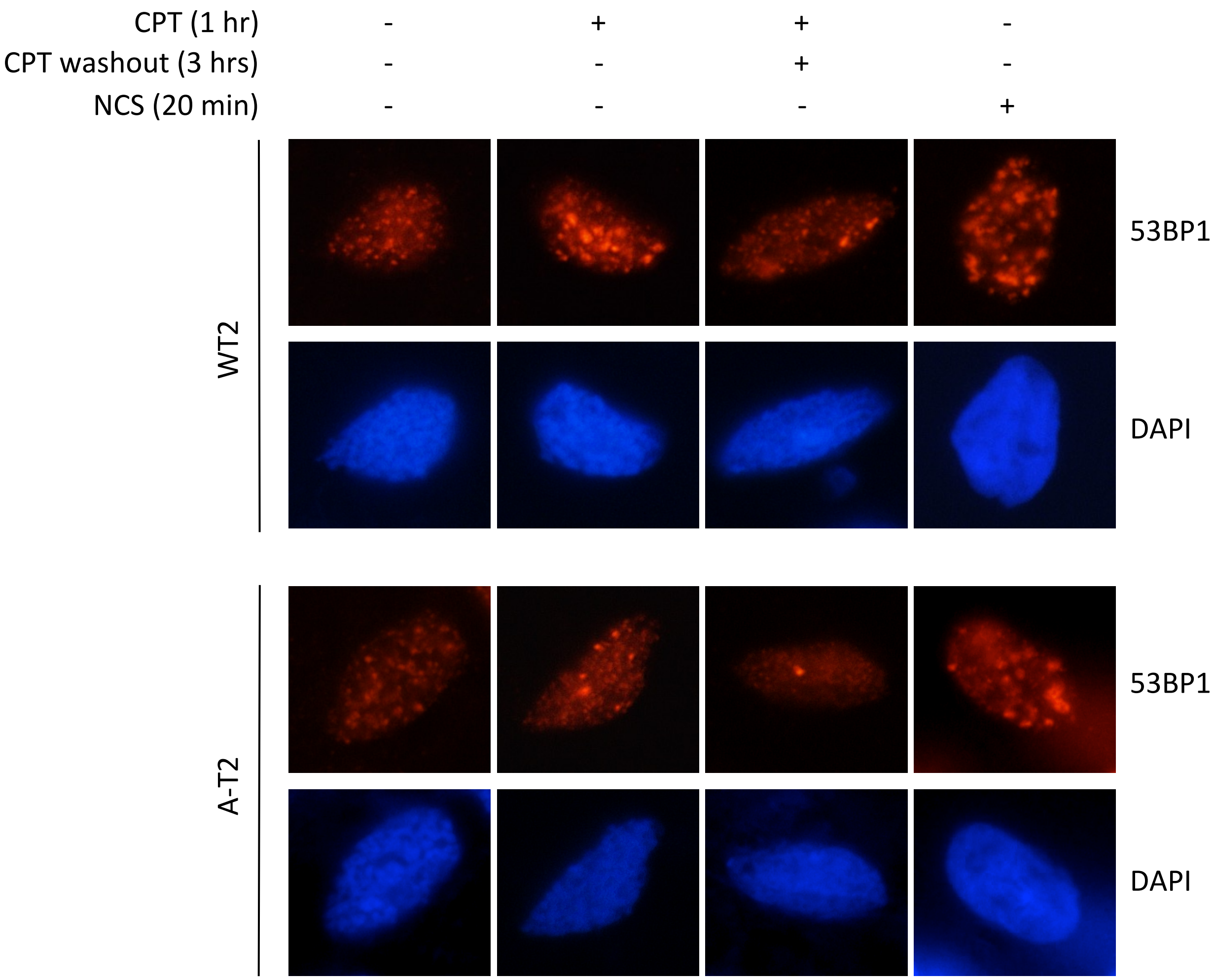

B

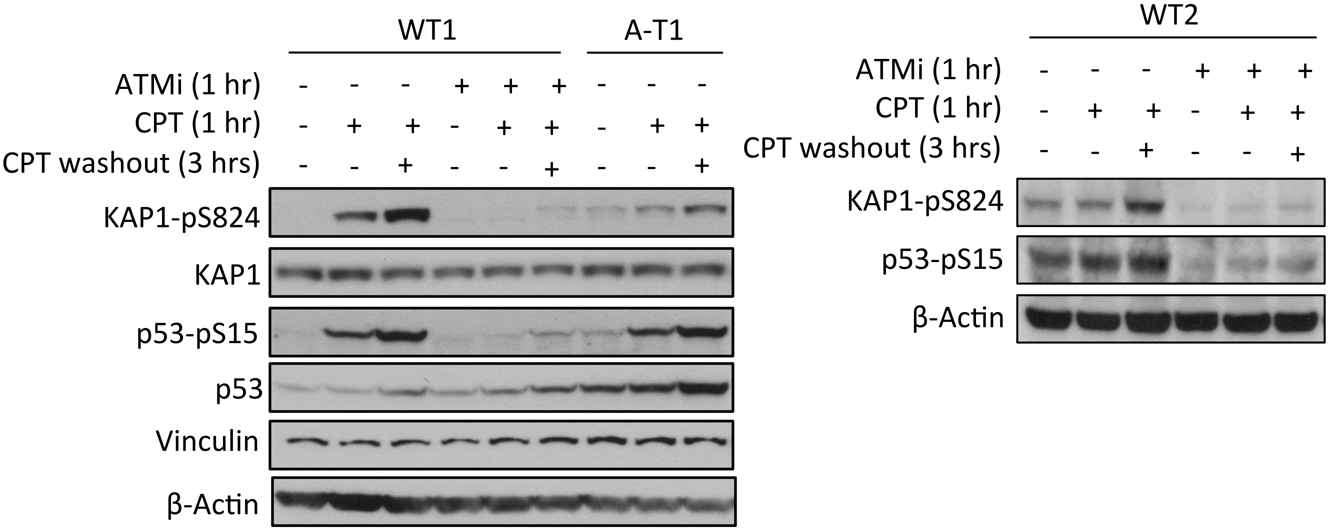

SUPPL. FIG 5

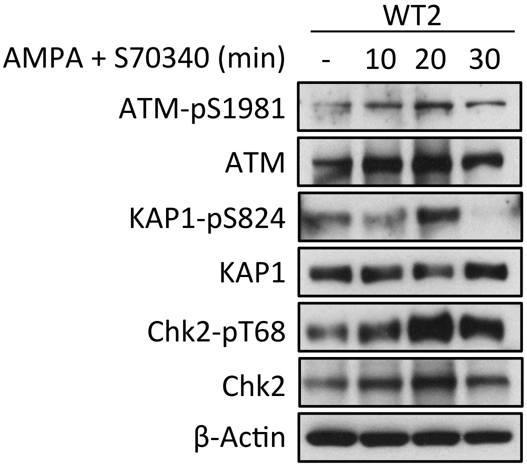
WT2
AMPA + S70340 (min)
-
10
20
30
ATM-pS1981
ATM
KAP1-pS824
KAP1
Chk2-pT68
Chk2
β-Actin

SUPPL. FIG 6

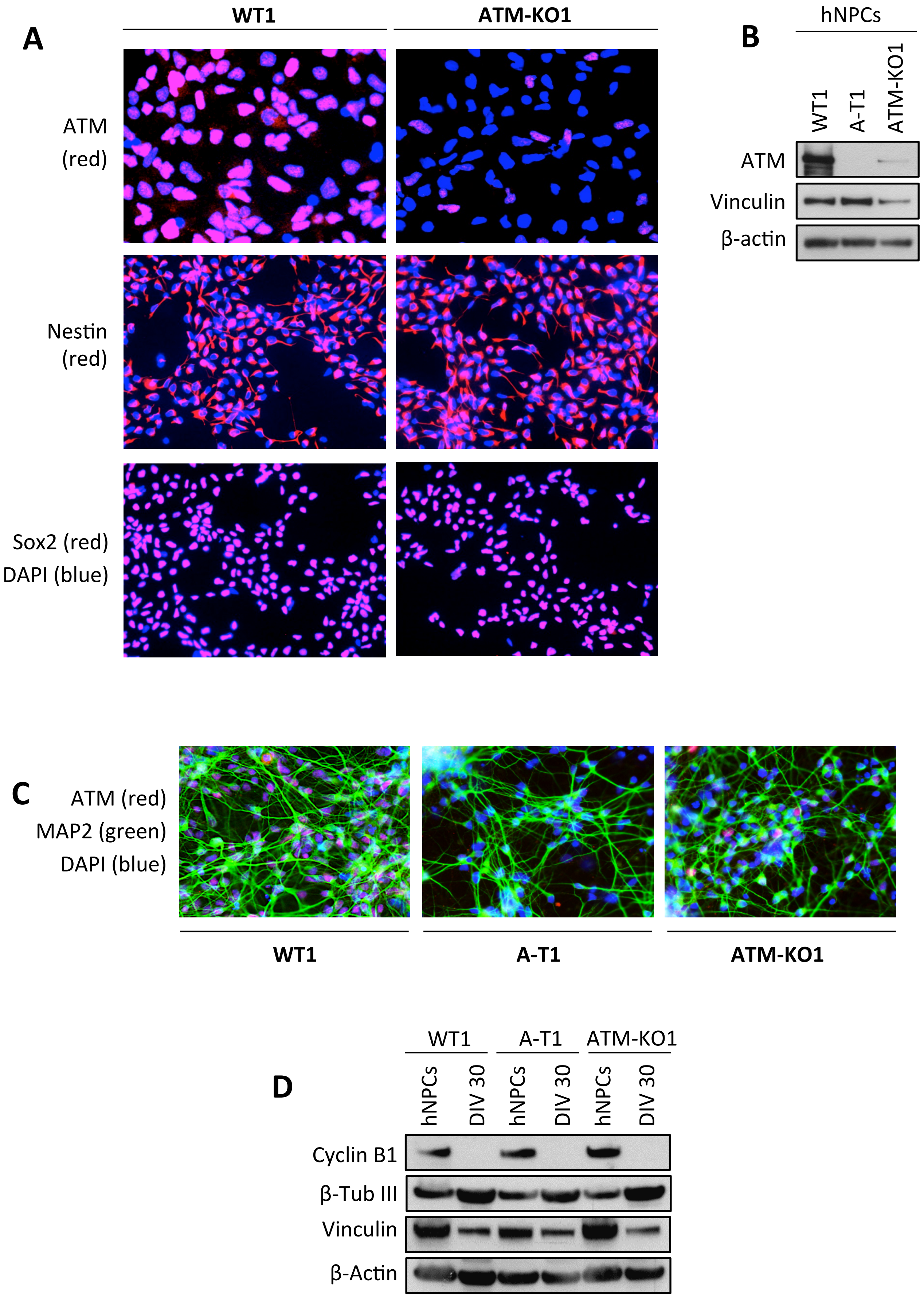
A
WT1
ATM-KO1
ATM
(red)
Nestin
(red)
Sox2 (red)
DAPI (blue)
B
hNPCs
WT1
A-T1
ATM-KO1
ATM
Vinculin
β-actin
C
ATM (red)
MAP2 (green)
DAPI (blue)
WT1
A-T1
ATM-KO1
D
WT1
A-T1
ATM-KO1
hNPCs
DIV 30
hNPCs
DIV 30
hNPCs
DIV 30
Cyclin B1
β-Tub III
Vinculin
β-Actin

SUPPL. FIG 7

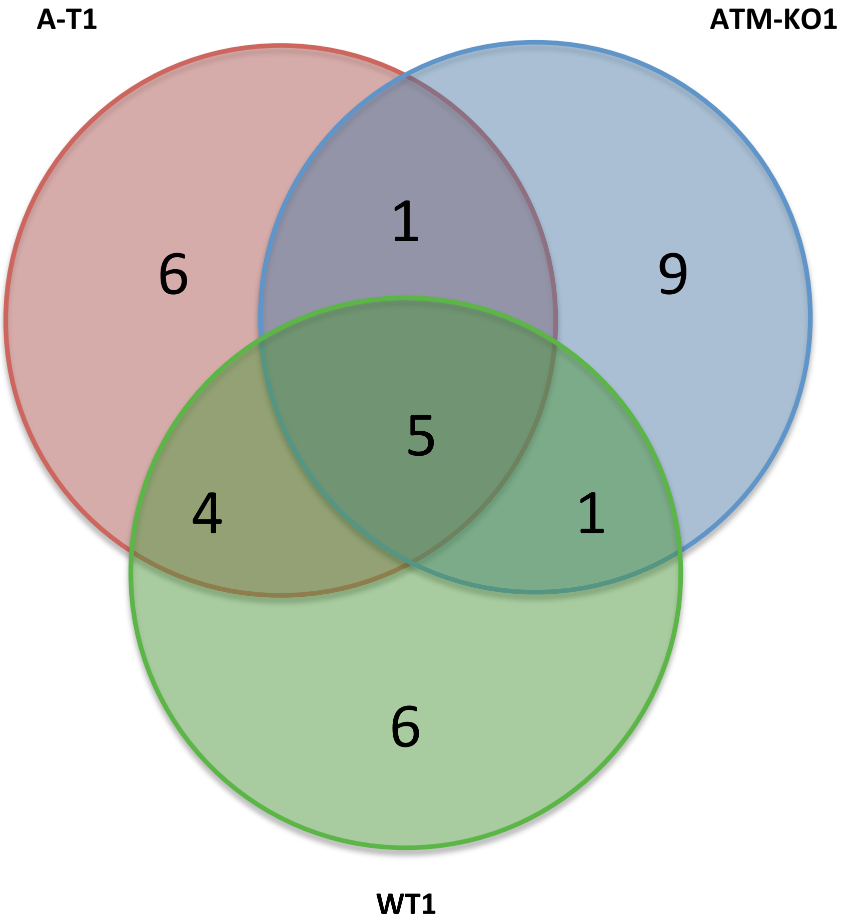

SUPPL. FIG 8

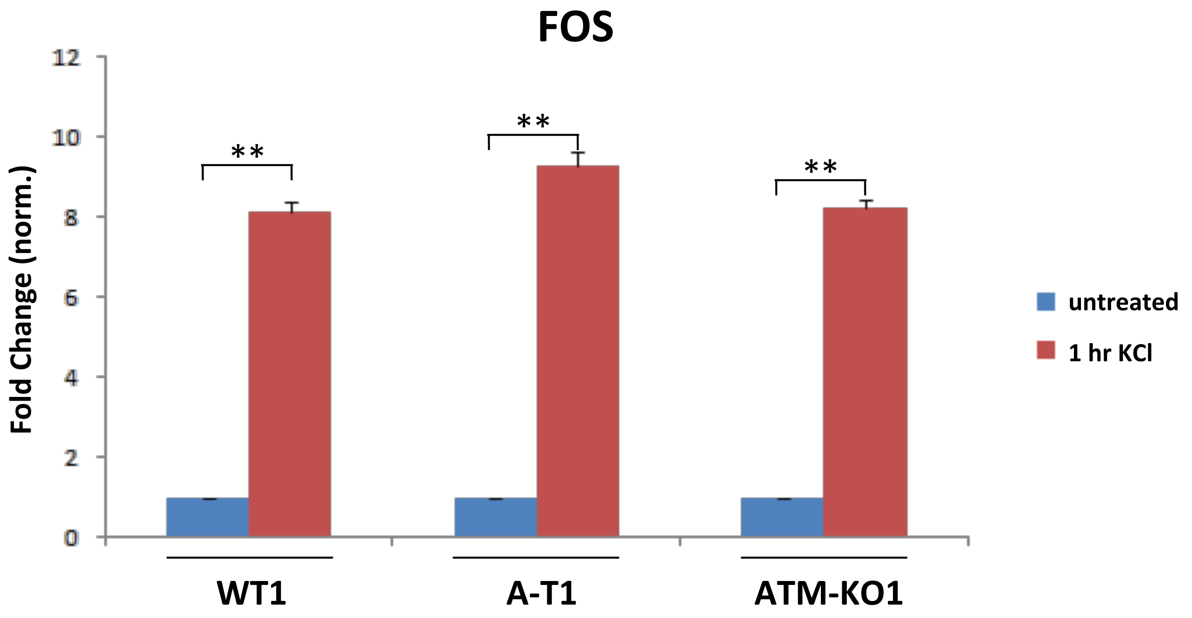
FOS
Fold Change (norm.)
0
2
4
6
8
10
12
**
**
**
untreated
1 hr KCl
WT1
A-T1
ATM-KO1

SUPPL. FIG 9

Upregulated genes

A

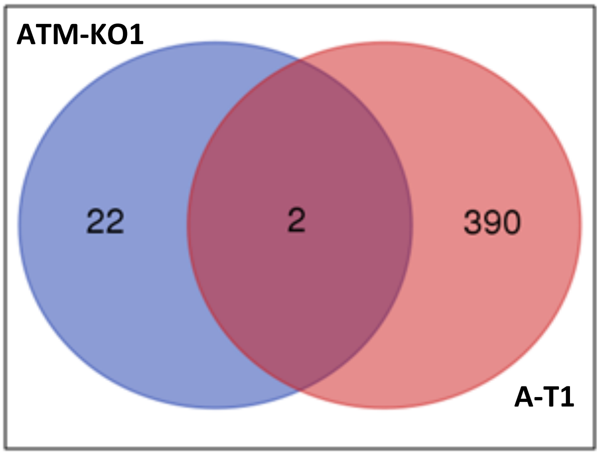

Downregulated genes

B

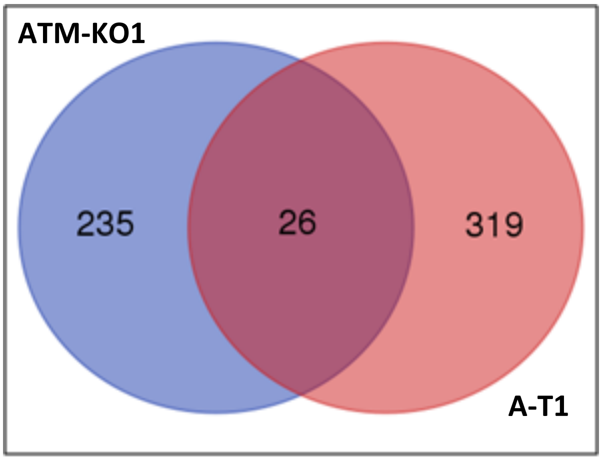

SUPPL. FIG 10
